# Mechanism of Ultra-Low-Speed Smoothness in Ultrasonic Motors Based on a Macro-Micro Multi-Scale Finite Element Model

**DOI:** 10.3390/mi17060659

**Published:** 2026-05-26

**Authors:** Weijun Zeng, Tong Xie, Qiaoliang Peng, Hengyu Zhang, Yifan Jiang, Lin Yang

**Affiliations:** 1 Rail Transit School, Zhejiang Institute of Communications, Hangzhou 311112, China; 2 State Key Laboratory of Mechanics and Control for Mechanical Structures, Nanjing University of Aeronautics and Astronautics, Nanjing 210016, China

**Keywords:** ultrasonic motor, superimposed pulse driving method, microstepping driving method, motor speed fluctuation rate, ADINA finite element simulation, ultrasonic friction reduction

## Abstract

The conventional microstepping driving method suffers from significant periodic speed oscillations under ultra-low-speed conditions, which fail to meet the stringent demand for smooth operation of ultrasonic motors in semiconductor packaging. Most existing theories and simulations of ultrasonic motors adopt a macroscopic mechanical perspective; after extensive linearization and idealization, they can only provide preliminary mechanism analysis and fail to achieve precise quantitative computation. Moreover, they neglect critical factors such as the microstructure of contact surfaces, preload distribution, and vibration mode transmission, making it difficult to reflect the true characteristics of the motor—including strong nonlinearity, multiphysics coupling, and complex interface behavior—resulting in considerable discrepancies between theory and experiment. In this paper, a macro-micro multi-scale finite element model of a traveling-wave ultrasonic motor is established using ADINA and HyperMesh, fully accounting for the strong nonlinearity and multiphysics coupling effects. Based on the ultrasonic friction reduction theory and the beat traveling wave mechanism, the stator deformation, interface zoning characteristics, and torque output of the superimposed pulse driving method and the microstepping driving method are systematically compared. The simulated stator mode shapes are validated by laser scanning vibrometry experiments, and multiple speed tests ranging from 200 to 320 arcsec/s are conducted. Simulation results show that at a target speed of 900 arcsec/s, the superimposed pulse driving method reduces the speed fluctuation rate from 228% to 32%. Experimental results confirm that the speed fluctuation rate of the superimposed pulse driving method is consistently much lower than that of the microstepping driving method across the entire tested speed range. This study reveals the low-speed smooth operation mechanism of the superimposed pulse driving method, characterized by single-peak dominance and smooth alternation between the driving and braking zones, thereby fundamentally overcoming the inherent shortcomings of the traditional microstepping driving method. The proposed model can effectively replace costly direct interface measurements, providing a new method and reference for ultra-low-speed precision control of ultrasonic motors and for investigating the driving mechanisms of similar motors.

## 1. Introduction

Semiconductor packaging equipment is the core foundation for the realization of advanced packaging technologies. The performance of its actuating mechanisms and positioning platforms directly determines the quality of the packaging process, imposing stringent requirements on the drive units for high-resolution motion and high-precision positioning [1,2]. Since packaging quality is highly susceptible to vibrations and shocks during motion, the motion smoothness of key processes such as bonding, pick-and-place, and alignment directly determines the packaging interconnection accuracy, structural reliability, and product yield [3,4]. Therefore, it is essential for the drive elements to output a continuous and stable angular velocity vector to ensure precision control of critical processes such as bonding and packaging [5]. As a novel piezoelectric driving element, the ultrasonic motor offers distinct advantages such as fast response, high displacement resolution, and power-off self-locking [6,7,8], demonstrating broad application prospects in critical fields including aerospace, precision guidance, and semiconductor precision manufacturing. Its high-resolution characteristics (down to the sub-nanometer level) and fast dynamic response make it an ideal choice for meeting the high-precision positioning demands of advanced packaging equipment [9]. Although the field of ultrasonic motor speed control places extremely high demands on speed stability, research specifically addressing ultra-low-speed control (below 1°/s) remains relatively limited. When a motor operates in this speed range, conventional control techniques struggle to achieve satisfactory performance. This challenge can be addressed by leveraging the unique low-speed operational characteristics of ultrasonic motors; consequently, researchers have proposed the microstepping driving method to achieve stable ultra-low-speed operation [10,11,12,13,14,15].

Chen et al. [10] systematically analyzed the microstepping characteristics of a traveling-wave ultrasonic motor based on a transient response model, revealing the influence of driving parameters on microstepping motion and providing theoretical support for high-precision microstepping operation of ultrasonic motors. Qu et al. [11] proposed a high-precision positioning control method for ultrasonic motors based on transient response characteristics and achieved superior control performance in a precision positioning system. Shi et al. [12] developed an optimization method for low-speed precision control of a butterfly-shaped linear ultrasonic motor, attaining more stable low-speed operation and positioning control performance in a high-precision positioning system. Takano et al. [13] employed a longitudinal-bending multilayered transducer with independent electrodes, independently controlling the amplitude and phase of longitudinal and bending vibrations, thereby suppressing the nonlinear input-output characteristics of an ultrasonic linear motor to 1/20 of that of conventional motors, achieving a positioning resolution of 20 nm and low-speed driving at 0.1 mm/s. Delibas and Koc [14] addressed the issues of friction noise and motion unevenness in piezoelectric ultrasonic motors during low-speed operation by establishing a mechanical model considering the motion characteristics of resonant motors, realizing smooth low-speed motion from 1 mm/s down to 0.001 mm/s with a positioning error of less than 10 nm. The aforementioned studies predominantly apply the microstepping driving method to high-precision positioning scenarios of ultrasonic motors, achieving satisfactory positioning control results. However, in the field of speed control, most existing research has only realized basic low-speed operation capability, while in-depth investigations into the periodic speed oscillations that occur during low-speed operation remain limited. Experimental results indicate that when the microstepping driving method is employed for ultra-low-speed operation of ultrasonic motors, measurements obtained using a high-precision encoder reveal significant periodic oscillations in motor speed [15], severely affecting speed stability.

To date, research addressing the issue of speed oscillations during ultra-low-speed operation of ultrasonic motors under the microstepping driving mode remains insufficient. In response to this issue, the author has focused on it as a key research direction. A detailed analysis and elaboration of the oscillation mechanism were provided in Ref. [15], and the superimposed pulse driving method was proposed in Ref. [16]. Extensive experimental results demonstrate that this method significantly reduces the speed fluctuation rate during ultra-low-speed motor operation, effectively improving speed stability. This method serves as a core extension and optimization of the microstepping driving method. Compared with traditional microstepping driving method, this method significantly reduces the speed fluctuation rate of the motor. Theoretical analysis and experimental observations indicate that the oscillations are caused by the excessively short deceleration phase in the stopping area of the microstepping driving method. Furthermore, because the rotor is an elastic component, the rapid speed decrease generates substantial friction, causing the rotor—made of a thin hard aluminum plate—to undergo torsional deformation along its axis and exhibit substantial torsional vibration in the stopping area.

To verify the validity of this theoretical analysis, the author conducted a detailed analysis in Ref. [16] from the perspective of friction dynamics. However, most existing theoretical and simulation studies on ultrasonic motors adopt a macroscopic mechanical perspective, treating the motor as a single mechanical mechanism for kinematic, dynamic, and equivalent circuit modeling. Such approaches involve extensive linearization and idealization of the system. Although they can preliminarily reveal the driving principles, they neglect key factors such as the microstructure of the contact surface, preload distribution, and vibration mode coupling. Consequently, they struggle to capture the true characteristics of ultrasonic motors—including strong nonlinearity, multiphysics coupling, and complex interface behavior—leading to discrepancies between theoretical calculations and experimental results and an inability to accurately describe the dynamic performance of the motor. For the reasons outlined above, this paper will conduct further research based on the theoretical work presented in Ref. [16].

The stator modes and the stator-rotor contact interface constitute the core operational region of ultrasonic motors, yet they represent the weakest link in macroscopic analysis. Macroscopic analysis alone cannot reveal the microscopic contact state. During experiments, the stator and rotor are in close contact, and the housing enclosure provides preload. As a result, obtaining data related to the contact state through experiments is both costly and difficult to measure. Currently, instruments capable of accurately measuring interfacial friction are relatively scarce and extremely expensive. Therefore, this study moves beyond the single-scale macroscopic analysis framework. Taking the stator modes and the stator-rotor contact interface as entry points, it combines macroscopic overall dynamics with microscopic interface behavior to establish a motor simulation model that more closely approximates actual conditions. Since the finite element method can solve most engineering problems involving complex structures, this paper employs the finite element software ADINA System 9.7.2 to perform dynamic simulations of the complete motor. Through three-dimensional modeling and multiphysics finite element simulations, a “magnifying glass”-style detailed analysis of the motor is conducted. At the macroscopic scale, the stator vibration modes, electromechanical coupling characteristics, and overall rotor motion patterns are characterized. At the microscopic scale, the stress distribution at the contact surface and the dynamic friction mechanisms are analyzed. This approach upgrades traditional macroscopic simplified models into multi-scale models that integrate macro- and micro-level analyses, thereby maintaining an overall understanding of the system while addressing the shortcomings of purely theoretical analyses regarding microscopic nonlinearities and interface behavior, and significantly improving model accuracy and prediction reliability. This paper will use a combination of macroscopic and microscopic approaches to reveal the driving mechanism by which the superimposed pulse driving method achieves a lower speed fluctuation rate compared with the traditional microstepping driving method.

## 2. Ultrasonic Friction Reduction Theory of Ultrasonic Motors

The micro-driving mechanism of an ultrasonic motor relies on the vibration of points on the surface of an elastic medium to achieve friction drive. Under the inverse piezoelectric effect of piezoelectric materials, vibration waves are excited on the stator surface, causing the surface points to move in elliptical trajectories. The motion of these points includes both horizontal and vertical components. Horizontal relative sliding generates heat or deformation on the friction surfaces, which directly affects the coefficient of friction. The vertical component of motion causes the rotor to float upward, and high-frequency vibrations lead to frequent separation of the contact surfaces. The average coefficient of friction decreases as the vibration frequency increases, giving rise to the phenomenon of ultrasonic friction reduction [17]. Therefore, the ultrasonic friction reduction between the stator and rotor of an ultrasonic motor results from the combined effects of both vertical and horizontal motions.

### 2.1. Horizontal Ultrasonic Friction Reduction Theory

#### 2.1.1. Vibration Model Analysis of Points on the Stator and Friction Material in the Horizontal Direction

The core driving mechanism of a traveling-wave ultrasonic motor is that, under ideal pure traveling wave excitation, surface particles of the stator form closed elliptical trajectories. The tangential velocity component parallel to the stator-rotor contact interface is the primary driving force for rotor motion. Under pure traveling wave conditions, the friction layers of the stator and rotor only make effective contact at the wave crests, where the tangential velocity of the particles is output in the horizontal direction, consistent with the direction of rotor motion. The driving method proposed in this paper does not generate an ideal pure traveling wave, but rather a mixed traveling-standing wave formed by the superposition of a traveling wave and a standing wave. This type of composite vibration introduces a more significant vibration component perpendicular to the stator-rotor contact interface, thereby exhibiting notable ultrasonic friction reduction characteristics. At the same time, the resultant velocity direction of the contacting stator particles forms a fixed inclined angle with the rotor motion direction, directly altering the frictional transmission characteristics at the stator-rotor interface. Therefore, a targeted contact vibration model must be established for analysis. When the direction of ultrasonic vibration is perpendicular to the stator-rotor contact interface, a significant ultrasonic friction reduction phenomenon occurs [18].

**Figure 1:**
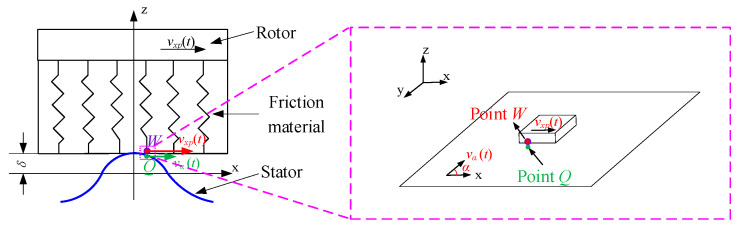
Schematic diagram of horizontal vibration analysis of the stator and friction material.

For convenience of analysis, a horizontal vibration model of the stator and friction material is established, as shown in Figure 1. A pair of contacting particles is selected in the stator-rotor contact area: point *Q* on the stator and point *W* on the friction material. Point *W* moves tangentially along the x-direction at a constant velocity $v_{xp}\left(t\right)$; the velocity of particle *Q* on the stator surface is $v_{\alpha }\left(t\right)$, with its direction forming an angle α with the x-axis. For convenience of analysis, a horizontal vibration model of the stator and friction material is established, as shown in Figure 1. A pair of contacting particles is selected in the stator-rotor contact area: point *Q* on the stator and point *W* on the friction material. Particle *W* moves tangentially along the x-direction at a constant velocity $v_{xp}\left(t\right)$; the velocity of particle *Q* on the stator surface is $v_{\alpha }\left(t\right)$, with its direction forming an angle α with the x-axis. The spring symbol in Figure 1 is used to characterize the equivalent normal contact stiffness of the friction material. Based on contact mechanics principles, the elastic compression behavior of the friction layer is simplified as a distributed linear spring to provide an intuitive mechanical representation of the normal contact pressure at point *W*. This spring-based equivalent modeling approach has been widely adopted in contact mechanics analyses of ultrasonic motors. It should be noted that Figure 1 is a geometric schematic for the horizontal vibration analysis, aimed at establishing the geometric relationships for the kinematic equations of the material point, rather than serving as a complete frictional mechanical model. The damping effects and nonlinear frictional behavior at the stator-rotor contact interface will be systematically analyzed in the following sections, in combination with the ultrasonic friction reduction theory, the pulsating friction force model, and the ADINA multi-scale finite element simulations.

#### 2.1.2. Kinematic Analysis of a Point in the Horizontal Direction

The following analysis is based on the premise that stator point *Q* and friction material point *W* are in contact and that velocities directed to the right are taken as positive. The kinematic analysis of point *W* on the surface of the friction material will be carried out under two scenarios.

##### When the Tangential Velocity of the Stator Point Is Greater than That of the Rotor Point and the Directions of Motion of the Two Points Are the Same, i.e., $v_{\alpha }\left(t\right)cos\alpha >v_{xp}\left(t\right)$ > 0

**Figure 2:**
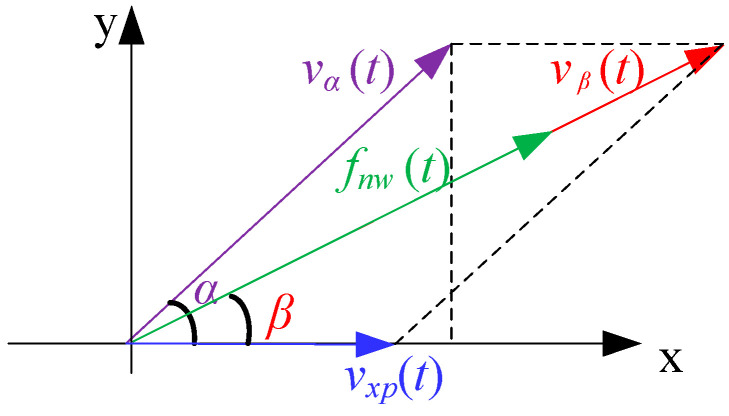
Type I vector diagram of velocity and friction force.

The rotor point moves tangentially along the x-direction with a velocity $v_{xp}\left(t\right)$. The tangential velocity of the stator surface point is $v_{\alpha }\left(t\right)$, forming an angle α with the x-axis. When the tangential velocity of the stator point is greater than that of the rotor point, the stator drives the rotor to accelerate through friction, and the direction of the friction force coincides with the direction of rotor motion. The kinematic analysis diagram of point *W* on the surface of the friction material is shown in Figure 2:

The resultant velocity $v_{\beta }\left(t\right)$ of point *W* is obtained by combining its own tangential motion velocity along the x-direction with the vibration velocity vector of stator point *Q*. The motion velocity of point *W* can be derived according to the velocity composition theorem in kinematics.

(1) $$v_{\beta }\left(t\right)=\sqrt{v_{\alpha }{\left(t\right)}^{2}+v_{xp}{\left(t\right)}^{2}+2v_{\alpha }\left(t\right)v_{xp}\left(t\right)\cos\alpha }\left(v_{\alpha }\left(t\right)\geq 0\right)$$

The angle between the ultrasonic vibration velocity $v_{\beta }\left(t\right)$ and the x-direction is β. The instantaneous friction force $f_{nw}\left(t\right)$ acting on point *W* is always opposite to the direction of the ultrasonic vibration velocity $v_{\beta }\left(t\right)$. The effective friction force $\bar{f_{mx}}\left(t\right)$, which resists the sliding motion of the rotor, is the average value of the projection of the instantaneous friction force onto the x-direction [19], and its expression is as follows:

(2) $$\bar{f_{mx}}\left(t\right)=\frac{1}{T_{jm}}\int_{0}^{T_{jm}}f_{nw}\left(t\right)\cos\beta dt$$

In Equation (2), $T_{jm}\left(t\right)$ represents the time required for stator point *Q* to complete one full elliptical motion, i.e., β can be derived based on the parallelogram law of vector composition and plane trigonometric relations.

(3) $$\beta =\arccos\left[\frac{1-\frac{v_{\alpha }\left(t\right)}{v_{xp}\left(t\right)}\cos\left(\alpha \right)}{{\left[1-2\frac{v_{\alpha }\left(t\right)}{v_{xp}\left(t\right)}\cos\left(\alpha \right)+{\left(\frac{v_{\alpha }\left(t\right)}{v_{xp}\left(t\right)}\right)}^{2}\right]}^{\frac{1}{2}}}\right],\left(v_{\alpha }\left(t\right)\geq 0\right)$$

##### When the Tangential Velocity of the Stator Point Is Less than That of the Rotor Point and the Directions of Motion of the Two Points Are the Same, i.e., $0<v_{\alpha }\left(t\right)cos\alpha <v_{xp}\left(t\right)$

**Figure 3:**
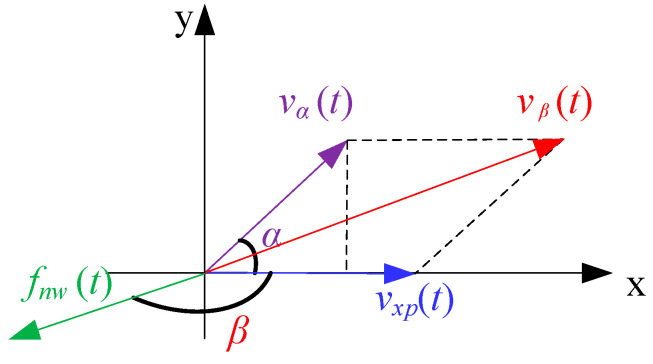
Type II vector diagram of velocity and friction force.

When the tangential velocity of the stator point is less than that of the rotor point, the direction of the interfacial friction force is opposite to the direction of rotor rotation, creating a hindering effect on the rotor motion. The stator relies on the interfacial friction force to suppress the rotation of the rotor. A kinematic analysis of point *W* on the surface of the friction material is conducted, as shown in Figure 3.

The motion velocity of point *W* is derived according to the velocity composition theorem in kinematics, and the resulting expression is identical to Equation (1). The instantaneous friction force $f_{nw}\left(t\right)$ acting on point *W* is always opposite to the direction of the ultrasonic vibration velocity $v_{\beta }\left(t\right)$. The effective friction force $\bar{f_{mx}}\left(t\right)$, which resists the sliding motion of the rotor, is defined as the average value of this instantaneous friction force, and its expression is exactly the same as Equation (2). In this equation, β can be calculated using the following formula, which is expressed as:

(4) $$\beta =\pi -\arccos\left[\frac{1-\frac{v_{\alpha }\left(t\right)}{v_{xp}\left(t\right)}\cos\left(\alpha \right)}{{\left[1-2\frac{v_{\alpha }\left(t\right)}{v_{xp}\left(t\right)}\cos\left(\alpha \right)+{\left(\frac{v_{\alpha }\left(t\right)}{v_{xp}\left(t\right)}\right)}^{2}\right]}^{\frac{1}{2}}}\right],\left(v_{\alpha }\left(t\right)\geq 0\right)$$

##### When the Tangential Velocity of the Stator Point Is Greater than That of the Rotor Point and the Directions of Motion of the Two Points Are the Same, i.e., $v_{\alpha }\left(t\right)cos\alpha <0\;<v_{xp}\left(t\right)$

**Figure 4:**
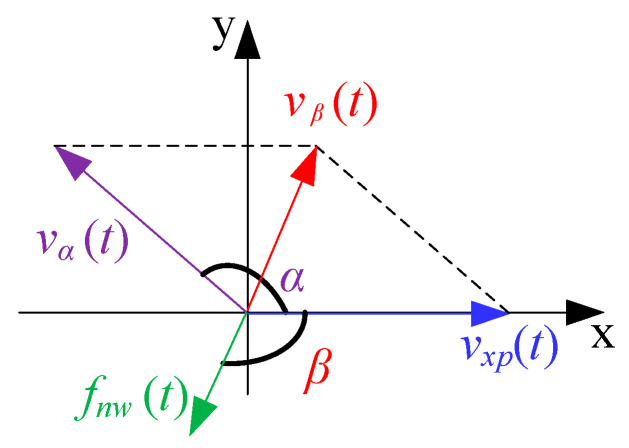
Type III vector diagram of velocity and friction force.

When the tangential velocity direction of the stator point is opposite to that of the rotor point, the direction of the interfacial friction force is opposite to the direction of rotor rotation, creating a hindering effect on the rotor motion. The stator relies on the interfacial friction force to suppress the rotation of the rotor. A kinematic analysis of point *W* on the surface of the friction material is conducted, as shown in Figure 4.

The resultant velocity $v_{\beta }\left(t\right)$ of point *W* is obtained by combining its own tangential motion velocity along the x-direction with the vibration velocity vector of stator point *Q*. The motion velocity of point *W* can be derived according to the velocity composition theorem in kinematics.

(5) $$v_{\beta }\left(t\right)=\sqrt{v_{\alpha }{\left(t\right)}^{2}+v_{xp}{\left(t\right)}^{2}-2v_{\alpha }\left(t\right)v_{xp}\left(t\right)\cos\left(\alpha \right)},\left(v_{\alpha }\left(t\right)<0,\alpha \in \left[0,\pi \right]\right)$$

The instantaneous friction force $f_{nw}\left(t\right)$ acting on point *W* is always opposite to the direction of the ultrasonic vibration velocity $v_{\beta }\left(t\right)$. The effective friction force $\bar{f_{mx}}\left(t\right)$ resists the sliding motion of the rotor is defined as the average value of this instantaneous friction force, and its expression is exactly the same as Equation (2). In this equation, β can be calculated using the following formula, which is expressed as:

(6) $$\begin{array}{l} \cos\beta =\pi +\arccos\left[\frac{1-\frac{v_{\alpha }\left(t\right)}{v_{xp}\left(t\right)}\cos\left(\alpha \right)}{{\left[1-2\frac{v_{\alpha }\left(t\right)}{v_{xp}\left(t\right)}\cos\left(\alpha \right)+{\left(\frac{v_{\alpha }\left(t\right)}{v_{xp}\left(t\right)}\right)}^{2}\right]}^{\frac{1}{2}}}\right],\left(v_{\alpha }\left(t\right)<0,\alpha \in \left[0,\pi \right]\right) \end{array}$$

### 2.2. Analysis of the Equivalent Coefficient of Friction

In Figure 1, the x-coordinate of point *W* on the surface of the friction material is $x_{w}$. Based on Hooke’s law and the fundamental contact mechanics model of ultrasonic motors [20], the expression for the pressure at point *W* is given by:

(7) $$p\left(x_{w},t\right)=k_{f}R_{sc}\varepsilon \left(\zeta_{z}\left(x_{w},t\right)-\delta_{0}\right)$$

where $k_{f}$ represents the equivalent stiffness of the contact area. The output friction can be calculated by the surface integral of the forces within the contact area. If the friction coefficient is defined as μ, ε means the width of the contact surface,$\;R_{sc}$ represents the value on the middle radius at the contact surface. $\zeta_{z}\left(x_{w},t\right)$ represents the instantaneous normal displacement of a stator point, which is a function of position $x_{w}$ and time t. $\delta_{0}$ denotes the initial preload deformation (static preload deformation), a constant representing the initial compression of the stator and friction material when stationary. When $\zeta_{z}\left(x_{w},t\right)>\delta_{0}$, the contact pressure $p\left(x_{w},t\right)>0$ and the interface is in contact; when $\zeta_{z}\left(x_{w},t\right)\leq \delta_{0}$, the contact pressure $p\left(x_{w},t\right)=0$ and the interface is in a separated state. The surface area of the friction material is denoted as $S_{wm}$. Based on the basic definition of pressure, the expression for the preload on the surface of the friction material is as follows:

(8) $$F_{cw}\left(x_{w},t\right)=p\left(x_{w},t\right)S_{wm}$$

According to the basic definition of Coulomb friction, the ratio of the effective friction force $\bar{f_{mx}}\left(t\right)$(Equation (2)) to the preload on the surface of the friction material $F_{cw}$ (Equation (8)) yields the expression for the equivalent coefficient of friction:

(9) $$\bar{\mu_{w}}\left(t\right)=\frac{1}{T_{jm}}\int_{0}^{T_{jm}}\frac{f_{nw}\left(t\right)}{F_{cw}}\cos\beta dt$$

### 2.3. Analysis of Instantaneous Friction Force

According to Coulomb’s law of friction, the sliding friction force acting in a direction parallel to the friction surface is proportional only to the normal force on the friction surface. The expression for the instantaneous friction force when point *W* of the friction material is in constant contact with point *Q* on the stator surface is shown below:

(10) $$f_{nw}\left(t\right)=\mu F_{cw}\left(x_{w},t\right)$$

When the vibration frequency of the stator is high, due to the inertia of the rotor, the rotor appears to hover above the stator [21] and cannot move up and down synchronously with the stator surface. Therefore, during high-speed vibration of the stator surface, the friction material of the stator and rotor are not in full contact throughout the entire motion. From a macroscopic perspective, it is difficult to observe a sudden change in the friction force. However, from a microscopic perspective, the contact state between the friction material of the stator and rotor alternates continuously between separation and contact. Consequently, the macroscopic friction force during vibration should be taken as the average value of the “pulsating” friction force $f_{nw}(t)$ shown in Figure 4 (indicated by the dashed line in Figure 4). In the figure, the average friction force $f_{nw}(t)$ is lower than the static contact friction force, which represents the ultrasonic friction reduction phenomenon in the vertical direction between the stator and the friction material. The expression for the average friction force for $N_{jm}$ separations between the friction material and the stator point [22] is as follows:

(11) $$\bar{f_{nw}}\left(t\right)=\frac{1}{\sum_{i=1}^{i=N_{jm}}T_{jmi}}\int_{0}^{\sum_{i=1}^{i=N_{jm}}T_{yi}}f_{nw}(t)dt,\left(i=1,2,3,\ldots \ldots \right)$$

In the above equation,$\;T_{jmi}(i=1,2,3,\ldots )$ represents the period of one complete up-and-down motion of stator point *Q*, where i denotes the number of cycles of the up-and-down motion; $T_{yi}$ represents the contact time between the stator point and the friction material during one cycle of the up-and-down motion; and $N_{jm}$ represents the number of times the stator point separates from the friction material. When the piezoelectric oscillator is in a state of longitudinal vibration, the oscillator momentarily separates from its contact surface during vibration, and the number of separations equals the vibration frequency. Due to the periodic nature of the vibration and its high frequency, a gap periodically appears between the piezoelectric oscillator and the contact surface, resulting in a significant reduction in the average coefficient of friction.

**Figure 5:**
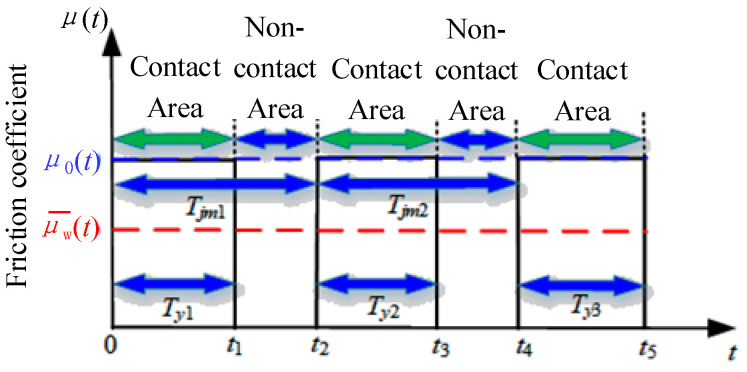
Schematic of the average coefficient of friction.

As shown in Figure 5, the friction force exhibits periodic variations, and the average coefficient of friction decreases (as indicated by the dashed line). This demonstrates that vertical vibration has a significant friction-reducing effect. $\bar{\mu_{w}}\left(t\right)$ is the average coefficient of friction of friction material point *W* over $N_{jm}$ cycles, and its expression, according to Coulomb’s law of friction, is as follows:

(12) $$\bar{\mu_{w}}\left(t\right)=\frac{1}{\sum_{i=1}^{i=N_{jm}}T_{jmi}}\int_{0}^{\sum_{i=1}^{i=N_{jm}}T_{yi}}\frac{f_{nw}(t)}{F_{cw}\left(x_{w},t\right)}dt,\left(i=1,2,3,\ldots \ldots \right)$$

### 2.4. Theoretical Analysis of Ultrasonic Friction Reduction Along Elliptical Trajectory

Both the stator of the ultrasonic motor and the points on the friction material vibrate in elliptical trajectories, thereby generating the ultrasonic friction reduction effect. Under high-frequency vibration of the stator, point *Q* and the friction material undergo periodic contact-separation alternation: tangential friction force is generated during contact, while the interface separates and the friction force becomes zero during separation. According to Coulomb’s law of friction, the expression [23] for the instantaneous tangential friction force of stator point *Q* is as follows:

(13) $$\mu_{wxz}\left(t\right)=\left\{\begin{array}{l} \mu_{0},\left(t\in \left[0,T_{yi}\right]\right)\\ 0,\left(t\in \left[T_{yi},T_{jmi}\right]\right) \end{array}\right.,\left(t\in \left[0,T_{jmi}\right],i=1,2,3,\ldots \ldots \right)$$

The instantaneous tangential friction force on stator point *Q* is

(14) $$f_{wxz}\left(t\right)=\left\{\begin{array}{l} \mu_{0}\left(t\right)F_{cw}\left(x_{w},t\right)\cos\beta ,\left(t\in \left[0,T_{yi}\right]\right)\\ 0,\left(t\in \left[T_{yi},T_{jmi}\right]\right) \end{array}\right.,\left(t\in \left[0,T_{jmi}\right],i=1,2,3,\ldots \ldots \right)$$

Substituting Equation (13) into Equation (11), the expression for the average tangential friction force over $N_{jm}$ cycles of elliptical motion is obtained as follows:

(15) $$\bar{f_{wxz}}\left(t\right)=\frac{1}{\sum_{i=1}^{i=N_{jm}}T_{jmi}}\int_{0}^{\sum_{i=1}^{i=N_{jm}}T_{yi}}\mu_{0}\left(t\right)F_{cw}\left(x_{w},t\right)\cos\beta dt$$

According to Coulomb’s law, the expression for the average equivalent coefficient of friction is as follows:

(16) $$\bar{\mu_{wxz}}\left(t\right)=\frac{1}{\sum_{i=1}^{i=N_{jm}}T_{jmi}}\int_{0}^{\sum_{i=1}^{i=N_{jm}}T_{yi}}\mu_{0}\left(t\right)\cos\beta dt$$

According to Newton’s third law, the interaction between stator point *Q* and friction material point *W* satisfies the action-reaction principle. Therefore, the instantaneous tangential friction force $f_{wxm}\left(t\right)$, the equivalent coefficient of friction $\bar{\mu_{wxm}}\left(t\right)$, and its average value $\bar{\mu_{wxmp}}\left(t\right)$ at point *W* are equal in magnitude but opposite in sign to those at point *Q*. That is:

(17) $$f_{wxm}\left(t\right)=-f_{wxz}\left(t\right)$$

(18) $$\bar{\mu_{wxm}}\left(t\right)=-\bar{\mu_{wxz}}\left(t\right)$$

(19) $$\bar{\mu_{wxmp}}\left(t\right)=-\bar{\mu_{wxzp}}\left(t\right)$$

### 2.5. Theoretical Analysis of Ultrasonic Friction Reduction at Contact Surfaces

According to tribological theory, sliding friction is a process that overcomes mechanical interlocking and molecular attractive forces of surface asperities; the friction force is the sum of mechanical and molecular resistive forces [24]. This is known as the friction binomial law, and its specific expression is as follows:

(20) $$f_{Ns}\left(t\right)=\tau_{c}S_{c}+\tau_{w}S_{w}$$

In the equation, $f_{Ns}\left(t\right)$ represents the sliding friction force at the contact surface; $S_{c}$ and $S_{w}$ denote the areas of molecular and mechanical interaction, respectively; and $\tau_{c}$ and $\tau_{w}$ represent the friction forces per unit area generated by molecular and mechanical interactions, respectively. Since the actual contact area $S_{0j}=S_{c}+S_{w}$, the expression for the friction force can be simplified to

(21) $$f_{Ns}\left(t\right)=\alpha_{c}S_{0j}+\beta_{c}F_{cs}$$

In the equation, $\alpha_{c}$ and $\beta_{c}$ are material-dependent constants, and $F_{cs}$ represents the preload. According to Coulomb’s law, the equivalent coefficient of friction for the contact area is:

(22) $$\mu_{s}\left(t\right)=\frac{\alpha_{c}S_{0j}}{F_{cs}}+\beta_{c}$$

Friction material serves as the primary medium through which torque is transmitted from the stator to the rotor. The preceding section focused on the equivalent coefficient of friction of points on the friction material surface. However, since the properties of individual points do not reflect the overall output characteristics of the motor, the following section will examine the equivalent coefficient of friction of the friction material surface as a whole.

**Figure 6:**
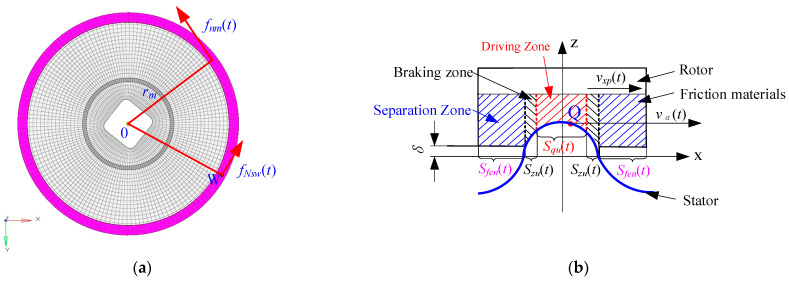
Analysis of the contact surface of the friction material. (**a**) Diagram of the contact surface of the friction material; (**b**) Zoning diagram of the contact interface friction material.

As shown in Figure 6, the total area of the friction material is defined as $S_{allm\;}$(the pink region in Figure 6a). At any given moment, within the region where the friction material is in contact with the stator, if the tangential velocity of a point is less than the tangential velocity at the constant-velocity point, this region is classified as the driving region (red region); otherwise, it is classified as the braking region (black region). Any region where the friction material is not in contact with the stator is defined in this paper as the separated region. The driving region represents the region where the tangential contact force is greater than zero, and the contact area between the stator and the friction material is denoted as $S_{qu}(t)$; the separated region represents the region where the tangential force is zero, and the contact area is denoted as $S_{fen}(t)$; the braking region represents the region where the tangential contact force is negative, and the contact area is denoted as $S_{zu}(t)$. In this paper, the total contact area between the friction material and the stator is defined as $S_{jcm}(t)$. Based on the above, let the area of any point *W* on the friction material be denoted as $S_{wm}.$ Combining Equations (8) and (21), the expression for the instantaneous tangential friction force at any point *W* on the friction material is given as follows:

(23) $$f_{Nsw}\left(t\right)=\alpha_{c}S_{\mathrm{sw}}+\beta_{c}F_{cw}\left(x_{w},t\right)$$

### 2.6. Theoretical Analysis of Motor Output Characteristics Based on the Ultrasonic Friction Reduction Theory

The frictional forces acting on a point *W* in the driving area (the red region in Figure 6b), the braking region (the black region in Figure 6b), and the separated region (the blue region in Figure 6b) are shown below:

(24) $$\left\{\begin{array}{l} f_{quw}\left(t\right)=sign\left(v_{xxw}\left(x_{w},t\right)-v_{xp}\left(t\right)\right)\left(\alpha_{c}S_{sw}+\beta_{c}F_{cw}\left(x_{w},t\right)\right)\\ f_{zuw}\left(t\right)=sign\left(v_{xxw}\left(x_{w},t\right)-v_{xp}\left(t\right)\right)\left(\alpha_{c}S_{sw}+\beta_{c}F_{cw}\left(x_{w},t\right)\right)\\ f_{fenw}\left(t\right)=0 \end{array}\right.$$

In the above equation, $v_{xp}(t)$ and $v_{\alpha }(t)$ represent the tangential velocities of friction material point *W* and stator point *Q* in contact with it, respectively, where

(25) $$sign\left(v_{\alpha }\left(x,t\right)-v_{xp}\left(t\right)\right)=\left\{\begin{array}{l} 1,\left(\left(v_{\alpha }\left(x,t\right)-v_{xp}\left(t\right)>0,v_{xp}\left(t\right)>0\right)\cup \left(v_{\alpha }\left(x,t\right)-v_{xp}\left(t\right)>0,v_{xp}\left(t\right)<0\right)\right)\\ -1,\left(\left(v_{\alpha }\left(x,t\right)-v_{xp}\left(t\right)<0,v_{xp}\left(t\right)>0\right)\cup \left(v_{\alpha }\left(x,t\right)-v_{xp}\left(t\right)<0,v_{xp}\left(t\right)<0\right)\right) \end{array}\right.$$

According to Coulomb’s law, the equivalent coefficient of friction for point *W* in each region of the friction material can be derived from Equation (24) as follows:

(26) $$\left\{\begin{array}{l} \mu_{quw}\left(t\right)=\frac{\alpha_{c}S_{sw}}{F_{cquj}\left(t\right)}+\beta_{c}\\ \mu_{zuw}\left(t\right)=\frac{\alpha_{c}S_{sw}}{F_{czuj}\left(t\right)}+\beta_{c}\\ \mu_{fenw}\left(t\right)=0 \end{array}\right.,\left(j=1,2,3,\ldots \ldots \right)$$

In the equation, $F_{cquj}\left(t\right)$ and $F_{czuj}\left(t\right)$ represent the preload on the contact surfaces of friction material point W in the driving region and the braking region, respectively, while $\mu_{quw}\left(t\right)$, $\mu_{zuw}\left(t\right)$, and $\mu_{fenw}\left(t\right)$ represent the equivalent friction coefficients of friction material point *W* in the driving region, the braking region, and the separated region, respectively. Here, j denotes the j point in each region.

Since motor output performance results from the combined effect of all points in the friction material, analyzing all points is necessary. Let the total number of points in the three regions of the friction material be $N_{allm}$. The numbers of points in the driving region, braking region, and separated region are $N_{qum}(t)$, $N_{zum}(t)$, and $N_{fenm}(t)$, respectively. The interfacial friction force in the driving region, braking region, and separated region is the sum of the forces contributed by the points in each region, expressed as

(27) $$\left\{\begin{array}{l} f_{qum}\left(t\right)=\sum_{j=1}^{j=N_{qum}\left(t\right)}\left(f_{quw}\left(t\right)\right)\\ f_{zum}\left(t\right)=\sum_{j=1}^{j=N_{zum}\left(t\right)}\left(f_{zuw}\left(t\right)\right)\\ f_{fenm}\left(t\right)=0 \end{array}\right.$$

The instantaneous tangential friction force acting at the interface of the friction material is the vector sum of three forces.

(28) $$f_{nm}\left(t\right)=f_{qum}\left(t\right)+f_{zum}\left(t\right)+f_{fenm}\left(t\right)$$

The average value of the tangential friction force acting at the interface of the friction material over $N_{jm}$ cycles of elliptical motion is

(29) $$\bar{f_{nm}}\left(t\right)=\frac{1}{\sum_{i=1}^{i=N_{jm}}T_{jmi}}\int_{0}^{\sum_{i=1}^{i=N_{jm}}T_{jmi}}f_{nm}\left(t\right)dt$$

Combining Equations (26), (27) and (29), the instantaneous equivalent coefficient of friction at the friction material interface is given by:

(30) $$\mu_{nm}\left(t\right)=\frac{\alpha_{c}S_{sw}\left(\sum_{j=1}^{j=N_{qum}(t)}\frac{1}{F_{cquj}(t)}-\sum_{j=1}^{j=N_{zum}(t)}\frac{1}{F_{czuj}(t)}\right)+\beta_{c}\left(N_{qum}(t)-N_{zum}(t)\right)}{N_{allm}}$$

Substituting Equation (30) into Equation (16) yields the average equivalent coefficient of friction for all points on the friction material interface over $N_{jm}$ cycles of elliptical motion.

(31) $$\bar{\mu_{nm}}\left(t\right)=\frac{1}{\sum_{i=1}^{i=N_{jm}}T_{jmi}}\int_{0}^{\sum_{i=1}^{i=N_{jm}}T_{jmi}}\mu_{nm}\left(t\right)dt$$

According to the torque theorem [25], the torque output by the motor at a given moment is

(32) $$M_{n}\left(t\right)=f_{nm}\left(t\right)\bar{r_{m}}$$

In the above equation, $r_{m}$ is the average radius of all points where the friction material contacts the stator, and its expression is

(33) $$\bar{r_{m}}\left(t\right)=\frac{\sum_{j=1}^{j=N_{allm}}r_{j}}{N_{allm}}$$

### 2.7. Limitations of Theoretical Model Analysis and Corresponding Solutions

The primary mechanism by which the superimposed pulse driving method facilitates low-speed motor operation is rooted in the beat traveling wave theory, characterized by periodic clockwise and counterclockwise rotational motion of stator points. Since the stator and rotor remain in contact during motor operation, the motion characteristics of points on the friction material surface are difficult to measure experimentally. Nevertheless, such data are critical for elucidating the underlying mechanism. Accordingly, the following section employs finite element analysis software to obtain the relevant data through simulation and performs a theoretical analysis based on the aforementioned formulas.

## 3. Parameter Settings for Finite Element Analysis Using ADINA Software

This paper employs ADINA (Automatic Dynamic Incremental Nonlinear Analysis) software and Altair HyperMesh 2022 software for finite element simulation. These tools offer powerful finite element solvers and preprocessing capabilities for mesh generation, respectively. ADINA is used for the finite element simulation analysis, which includes the mesh model of the entire motor, material models, damping models, contact surface settings, as well as load and boundary condition settings. The contact surface settings, material models, and damping models have been detailed in Ref. [26]. Since the model setup method used in this paper is consistent with that described in the reference, it will not be elaborated upon here.

### 3.1. Setting of Mesh Model and Model Loads

**Figure 7:**
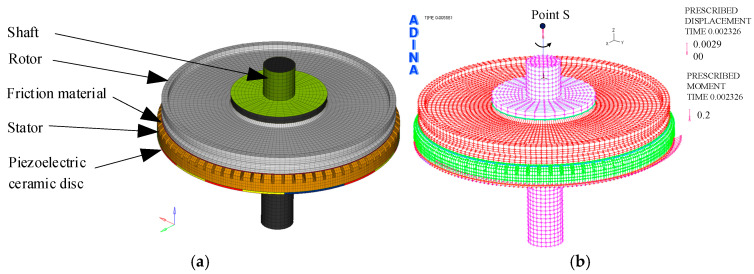
Three-dimensional mesh model of the complete motor. (**a**) Three-dimensional mesh model of the complete motor based on HyperMesh; (**b**) Three-dimensional simulation and torque load of the setting model complete motor based on ADINA; In (**b**), the colored parts are as follows: the red part represents the rotor, the green part represents the stator, and the purple part represents the shaft.

Figure 7 shows the three-dimensional simulation model of the complete motor, which consists of two parts: Figure 7a,b. Figure 7a presents the three-dimensional mesh model of the complete motor created using HyperMesh software, which is entirely composed of regular first-order hexahedral mesh elements. The stator mesh contains 86,778 elements, the friction material mesh contains 5184 elements, the piezoelectric ceramic ring mesh contains 6912 elements, the rotor mesh contains 67,680 elements, and the shaft mesh contains 23,080 elements. The model has a total of 249,824 nodes and 189,634 elements. The configuration of model loads and boundary conditions mainly consists of three parts: electrical excitation of the piezoelectric ceramic, torque loading on the rotating shaft, and preload application on the stator. Specifically, the torque load is applied to the rigid point *S* connected to the rotating shaft, as shown in Figure 7b, with a magnitude of 0.2 N·m. Thus, the torque load and the corresponding boundary conditions are defined.

**Figure 8:**
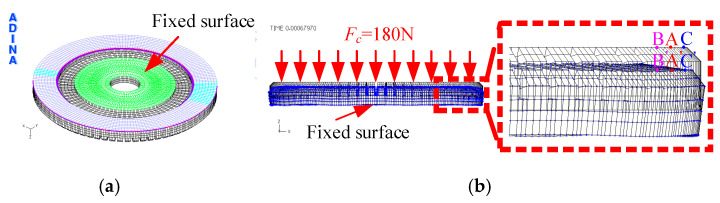
Fixed surface and deformation diagram of the stator. (**a**) Schematic diagram of the fixed surface of the stator; (**b**) Deformation diagram of the stator under preload.

Before applying the preload, a fully fixed constraint is first imposed on the stator. Figure 8a presents a schematic diagram of the fixed surface of the stator, which is intended only to visually indicate the constrained positions. On the basis of this constraint, a preload of 180 N is applied to the top surface of the stator. Since the stator is an elastic body, it undergoes elastic deformation under the preload, with the deformation on the outer circumference being larger than that on the inner circumference. The deformation patterns of points A, B, and C on the stator under this preload are illustrated in Figure 8b.

#### 3.1.1. Electrical Excitation Settings for the Mesh Mode

To reveal the mechanism by which the superimposed pulse driving method maintains a low speed fluctuation rate, subsequent simulation analyses in this paper require that the torque load and preload settings of the two driving methods remain consistent. Since the driving mechanisms of the two methods differ, their driving parameters also differ. By adjusting the driving parameters, the motors under both methods are set to achieve similar target speeds, with the parameter values for each method specified in Table 1 and Table 2, respectively. Specifically, the relevant parameters for the superimposed pulse driving method are listed in Table 1, including the driving frequency ($f_{d}$), the number of single-phase output pulses ($m_{s}$), and the number of pulses per cycle ($n_{p}$). The relevant parameters for the micro-step driving method are listed in Table 2, including the driving frequency ($f_{d}$), the number of pulses in the starting area ($m_{b}$), and the number of pulses in the stopping area ($n_{e}$). The specific definitions of the various pulse numbers have been detailed in Ref. [15] with figures and texts, and will not be repeated here. Only when the speed fluctuation rates are compared under similar target speeds can the differences between the two driving methods be effectively revealed. In the following sections, both driving methods will be used to conduct theoretical model analysis and ADINA full-system model analysis, respectively.

**Figure 9:**
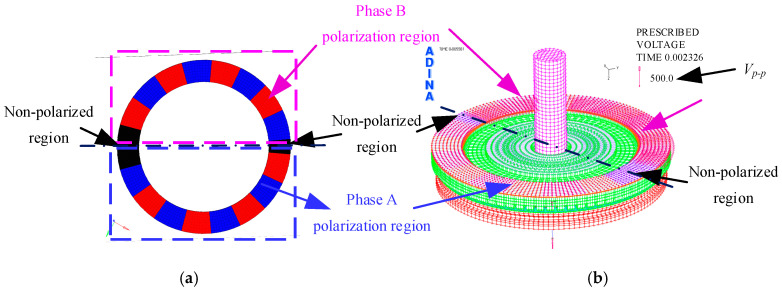
Polarization setting of the ceramic plate based on the complete motor model. (**a**) Polarization setting of the ceramic plate based on HyperMesh; (**b**) Polarization configuration of the ceramic plate based on ADINA.

The piezoelectric ceramics used in traveling-wave rotary ultrasonic motors are typically divided into polarized and non-polarized regions. The polarized region is further subdivided into two zones, A and B, separated by the non-polarized region. The piezoelectric material is configured as anisotropic, meaning that the two phases of the piezoelectric ceramic are divided into multiple polarized regions with adjacent forward and reverse polarizations. The polarization configuration of the ceramic plate is illustrated in Figure 9a. In ADINA, the voltage value is determined by setting the peak-to-peak voltage $V_{p-p}$ shown in Figure 9b. In the simulation, the piezoelectric ceramic is set as an anisotropic material, forming multiple alternating forward and reverse polarization regions. Figure 9a shows the polarization zone division model of the piezoelectric ceramic established using HyperMesh. After importing this model into ADINA software, the polarization properties were matched and configured, resulting in the corresponding piezoelectric ceramic simulation model. In the complete motor simulation model built with ADINA, the excitation voltage magnitude of the piezoelectric ceramic is determined by setting the peak-to-peak voltage $V_{p-p}$ = 500 V, as shown in Figure 9b.

**Figure 10:**
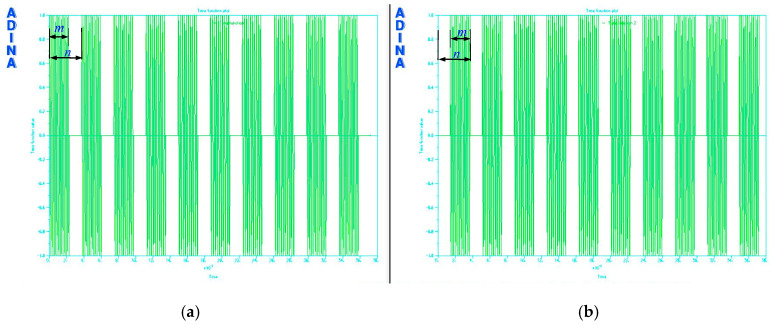
Two-phase voltage drive waveforms of the simulation model based on the superimposed pulse driving method in the ADINA environment. (**a**) Voltage drive waveform of phase A; (**b**) Voltage drive waveform of phase B.

**Figure 11:**
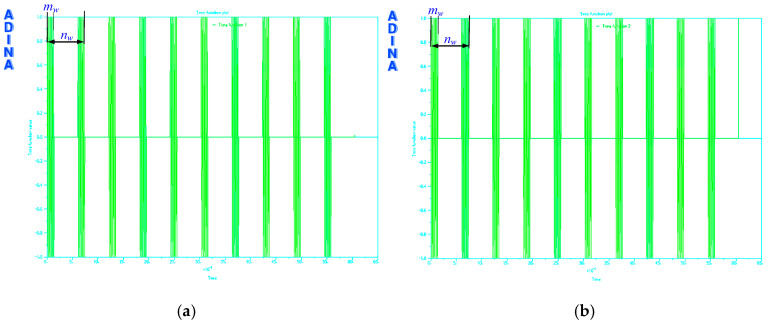
Two-phase voltage drive waveforms of the simulation model based on the microstepping driving method in the ADINA environment. (**a**) Voltage drive waveform of phase A; (**b**) Voltage drive waveform of phase B.

In the ADINA simulation model, the superimposed pulse driving method and the microstepping driving method were respectively applied to provide the driving excitation. The voltage drive waveforms of phases A and B of the piezoelectric ceramic plate are shown in Figure 10 and Figure 11, respectively.

#### 3.1.2. Simulation of Stator Modes

**Figure 12:**
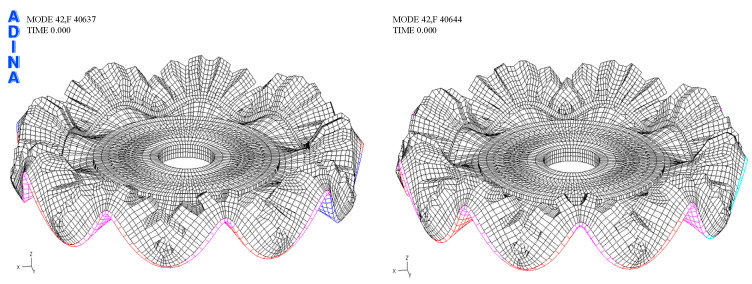
Mode shape diagram of the stator B09 mode from ADINA simulation.

The modal characteristics of the stator are critical to the overall performance of the motor. First, an 80 $V_{p-p}$ voltage was applied to the piezoelectric ceramics, and the stator base was fully constrained. A modal simulation was then performed on the USM-60 stator equipped with piezoelectric ceramics (supplied by Nanjing Hangda Super Control Technology Co., Ltd., Nanjing, China). The modal simulation results are presented in Figure 12, which shows the in-plane B_09_ mode with two identical mode shapes, each featuring nine peaks [27]. The corresponding natural frequencies are 40,637 Hz and 40,644 Hz, respectively.

### 3.2. Kinematic and Dynamic Simulation and Analysis

#### 3.2.1. Setting of Points on the Surfaces of the Stator and Friction Material

**Figure 13:**
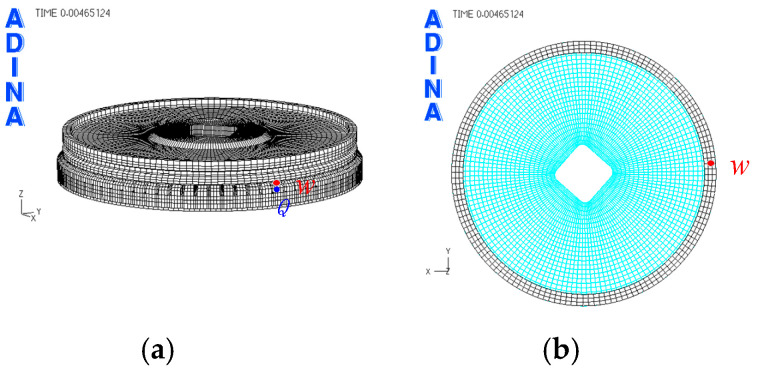
Schematic diagram of measurement points on the surfaces of the stator and friction material. (**a**) Definition of measurement points on the stator and friction material; (**b**) Arrangement of measurement points on the friction material surface.

The motion characteristics of each point on the friction material surface affect the output performance of the motor. In the ADINA simulation model, a point *W* on the friction material is arbitrarily selected, and its equivalent friction coefficient is taken as the focus of investigation. The corresponding point on the stator that contacts the friction material is denoted as point *Q*, as illustrated in Figure 13:

#### 3.2.2. Kinematic and Dynamic Simulation Based on the Superimposed Pulse Driving Method

##### Kinematic and Dynamic Simulation of Points

(1) Kinematic Simulation Analysis of Stator Point *Q*

**Figure 14:**
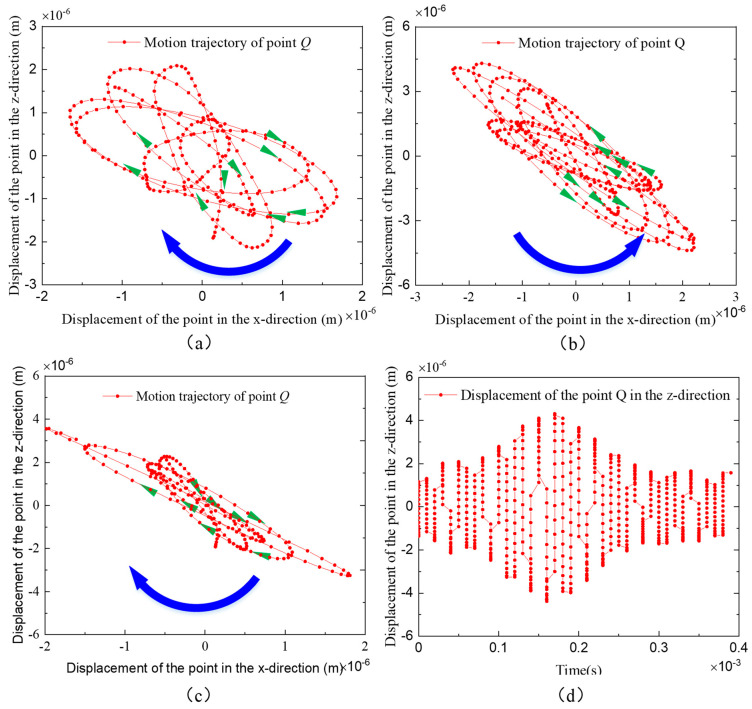
(**a**–**d**) Trajectory and z-direction displacement of stator point *Q*.

Based on the mechanism of the superimposed pulse driving method [16], each operating cycle can be divided into three stages: the traveling-standing wave amplitude reduction region ($T_{addT}$), the traveling-standing wave amplitude increase region ($T_{reduT}$), and the traveling-standing wave amplitude increase–decrease transition region ($T_{addreduT}$). Within one operating cycle, the motion direction of stator points alternates periodically between clockwise and counterclockwise, and the displacement exhibits an obvious beat traveling wave vibration characteristic. In the simulation model, point *Q* is selected as a monitoring point for dynamic simulation analysis. When the motor speed reaches a steady state, the motion trajectory of point *Q* is shown in Figure 14: the motion direction of the point sequentially changes from clockwise to counterclockwise and then to clockwise again, and the displacement response also exhibits a beat traveling wave pattern. The simulation results are in full agreement with the theoretical model derived in Ref. [16], thereby validating the correctness of the beat traveling wave theory underlying the superimposed pulse driving method. This also lays a foundation for subsequent dynamic simulation analysis of points.

(2) Dynamic Simulation of Point *W* on the Friction Material

**Figure 15:**
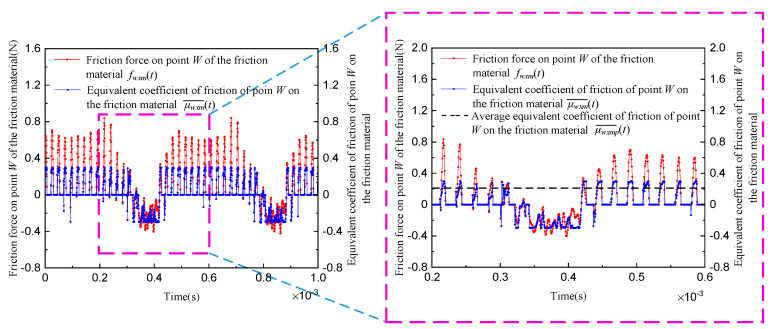
Analysis diagram of friction force and coefficient of friction for point W on the friction material under excitation of the superimposed pulse driving method.

When the motor model is based on the superimposed pulse driving method, the friction material point *W* and the stator point *Q* are in contact, and the motion of point *W* is driven by point *Q*. Due to their interaction, the direction of the friction force acting on point *W* changes periodically (as shown in the pink box in Figure 15). Furthermore, the high-frequency vertical vibration of stator point *Q* causes the rotor to experience a “floating” phenomenon, leading to alternating contact and separation between point *W* and point *Q*, which in turn results in an intermittent friction force. By combining Equations (17)–(19), the instantaneous tangential friction force $f_{wxm}\left(t\right)$, the equivalent coefficient of friction $\bar{\mu_{wxm}}\left(t\right)$, and its average value $\bar{\mu_{wxmp}}\left(t\right)$ for point *W* can be obtained. Substituting the motion simulation data of the point into Equation (19) yields an average equivalent coefficient of friction $\bar{\mu_{wxmp}}=0.01$9.

##### Kinematic and Dynamic Simulation of the Interface Between the Stator and the Friction Material

(1) Simulation Analysis of Stator Deformation and Contact Interface Stress of the Friction Material for the Complete Motor Model

**Figure 16:**
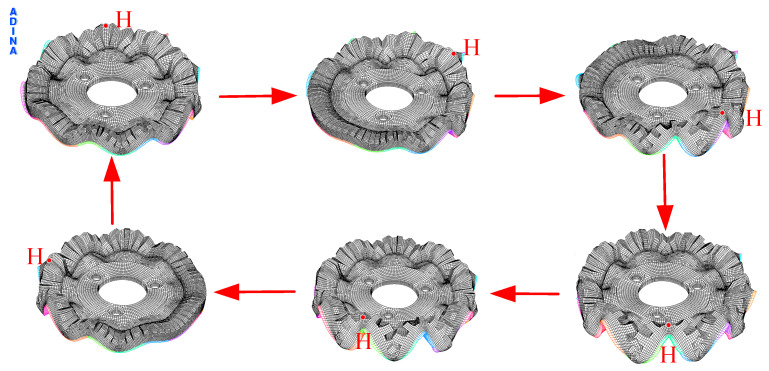
Schematic diagram of stator deformation under excitation of the superimposed pulse driving method.

Based on the friction binomial law, when the preload applied to the motor remains constant, changes in the contact area lead to variations in the equivalent coefficient of friction between the stator and the friction material, thereby affecting the output performance of the motor. Contact analysis of the interface was performed using the stator deformation and contour plot functions of ADINA [28]. The stator deformation plot based on the superimposed pulse driving method is shown in Figure 16.

**Figure 17:**
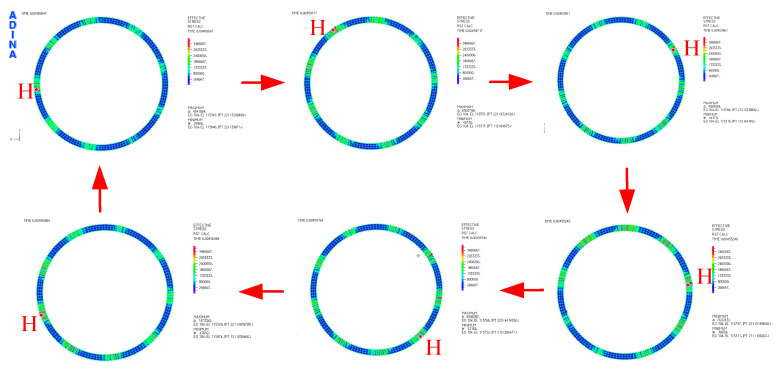
Stress distribution diagram of the stator-friction material contact interface under excitation of the superimposed pulse driving method.

Figure 16 reveals that, as the $B_{09}$ mode serves as the operating mode of the motor, the stator surface exhibits nine peaks. When the motor is driven by the superimposed pulse driving method, the amplitudes of the nine peaks are non-uniform. Among them, one peak attains the maximum amplitude (labeled as point H in the Figure), while the amplitudes of the remaining peaks diminish progressively on either side of the highest peak. In Figure 17, “△” indicates the location where the maximum effective stress (MAXIMUM) occurs, and “*” indicates the location where the minimum effective stress (MINIMUM) occurs. Notably, point H in Figure 16 and Figure 17 corresponds to the same MAXIMUM location.

As shown in Figure 16 and Figure 17, after preload is applied to the stator, the preload distribution on the surface of the rotor friction material exhibits a non-uniform characteristic. The wave crest with the largest amplitude on the stator surface (i.e., the wave crest labeled point *H* in the Figures) has a larger contact area with the friction material, resulting in a higher preload in this region and thus playing a dominant role in the driving process. The remaining wave crests with smaller amplitudes maintain larger gaps with the friction material, resulting in extremely small effective contact areas; accordingly, the preload and driving force in these regions are significantly weaker and cannot generate effective driving. In brief, among the nine wave crests on the stator, only a single high-amplitude wave crest assumes the core driving role, while the other crests exhibit either weak or no effective contact. This overall driving characteristic, dominated by a single point with multiple points contributing only weakly, reduces the upper limit of rotational speed at the source.

**Figure 18:**
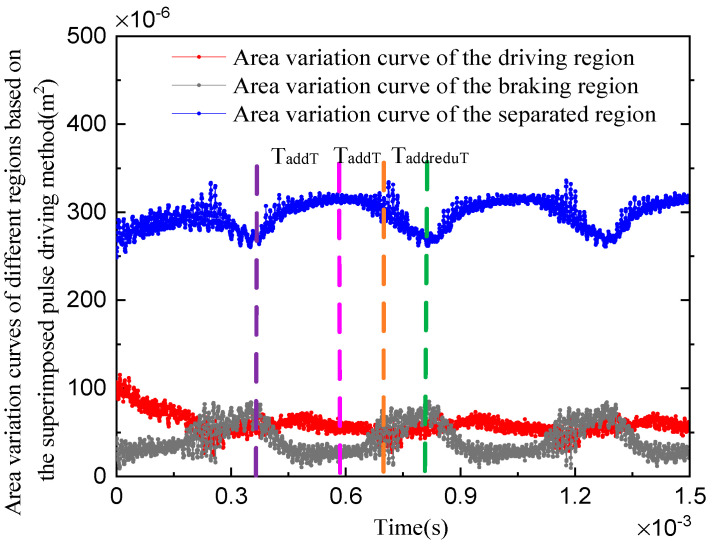
Area distribution diagrams of the driving region, braking region, and separated region based on the superimposed pulse driving method.

A single operating cycle of the superimposed pulse driving method consists of three stages: $T_{addT}$, $T_{reduT}$, and $T_{addreduT}$ [16]. Within any stage, the contact interface between the stator and rotor can be classified into three types according to their mechanical action characteristics: the region that provides forward driving force to the rotor is the driving region; the region that generates braking force on the rotor is the braking region; and the region where the stator and rotor are not in contact and no mechanical action occurs is the separated region. As shown in Figure 18, the areas of the driving, braking, and separated regions exhibit wave-like variations. The areas of the driving and braking regions fluctuate alternately with small amplitudes, occasionally showing the driving region slightly larger than the braking region or vice versa, while the area difference between them remains consistently balanced. According to Equation (24), the magnitudes of both driving force and braking force are proportional to the contact area of the corresponding region: the larger the driving region, the stronger the driving force; the larger the braking region, the stronger the braking force. Over a complete cycle of the superimposed pulse driving method, the total area of the driving region is slightly larger than that of the braking region. Consequently, the motor speed exhibits only mild fluctuations, accelerating slightly at times and decelerating slightly at others, while the rotor consistently rotates in a single direction without any reversal. Furthermore, the separated region occupies a much larger area than the other two regions. Since no effective mechanical action occurs in this region, energy input is substantially reduced, thereby enabling low-speed motor operation from a mechanistic perspective. The coordinated interaction of the three regions, with their smooth alternating transitions, forms the core mechanism that achieves stable ultra-low-speed motor operation.

(2) Simulation Analysis of Friction Force and Coefficient of Friction at the Friction Material Interface under excitation of the superimposed pulse driving method

**Figure 19:**
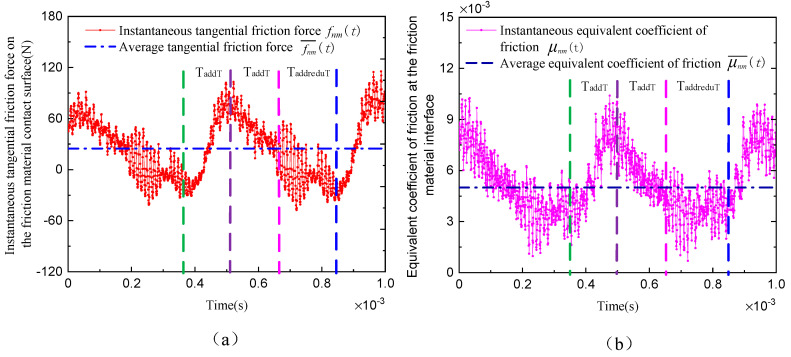
Analysis of friction forces and friction coefficients at the friction material interface under excitation of the superimposed pulse driving method. (**a**) Instantaneous and average friction forces at the interface of friction material; (**b**) Instantaneous and average equivalent coefficients of friction at the friction material interface.

Using Equations (28)–(31), after the motor speed has stabilized, the instantaneous tangential friction force $f_{nm}(t)$ on the friction material contact surface and its average over $N_{jm}$ cycles of elliptical motion, $\bar{f_{nm}}(t)$, are shown in Figure 19a. The instantaneous equivalent coefficient of friction $\mu_{nm}(t)$ at the friction material interface and its average over $N_{jm}$ cycles, $\bar{\mu_{nm}}(t)$, are presented in Figure 19b.

According to Equation (29), the average tangential friction force at the friction material interface over $N_{jm}$ cycles of elliptical motion is given by $\bar{f_{nm}}$ = 24 N. According to Equation (31), the average equivalent friction coefficient at the friction material interface over $N_{jm}$ cycles of elliptical motion is given by $\bar{\mu_{nm}}$ = 0.005.

The regional mechanical characteristics of the contact interface and the dynamic variations in the tangential friction force and equivalent coefficient of friction directly determine the torque output characteristics and speed behavior of the complete motor. To further reveal the overall operational performance of the motor under the superimposed pulse driving method, output performance simulations are conducted based on the complete motor finite element model, with a focus on the dynamic response of torque and speed.

(3) Simulation Analysis of Output Performance of the Complete Motor Model under excitation of the superimposed pulse driving method

**Figure 20:**
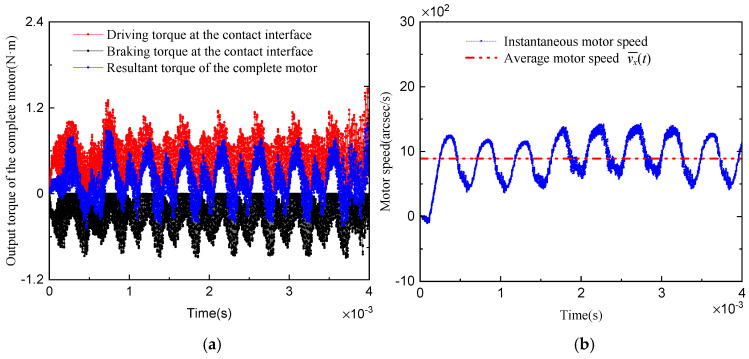
Simulation analysis diagram of motor torque output and speed based on the superimposed pulse driving method. (**a**) Simulation analysis diagram of motor torque output; (**b**) Simulation analysis diagram of motor speed.

To further reveal the influence of the mechanical action differences among the driving region, braking region, and separated region on the output characteristics of the complete motor, the torque of each region and the speed variation in the complete motor are obtained through finite element simulation, as shown in Figure 20.

Based on Equation (32), the simulation curves of torque for the three regions and motor speed are shown in Figure 20a, which illustrates the torque characteristics of the three regions: the driving region provides driving torque, the braking region generates braking torque, and the separated region produces no effective torque. Under the combined effect of the three regions, the overall output torque of the motor exhibits wave-like fluctuations, which in turn cause the instantaneous speed to vary regularly over time. The target motor speed is set to ${v_{tar}\left(t\right)=9\times 10}^{2}$ arcsec/s. Under the conditions of applied preload and load, the simulated motor speed curve obtained using ADINA software under the superimposed pulse driving method is shown in Figure 20b. The simulated average motor speed is ${\bar{v_{x}}\left(t\right)=8.92\times 10}^{2}$ arcsec/s, which is in good agreement with the target speed, and the speed fluctuation rate is 32%.

#### 3.2.3. Kinematic and Dynamic Simulation Based on the Microstepping Driving Method

Ref. [16] shows that when a motor is driven by the microstepping driving method, the rotational speed exhibits significant large-amplitude periodic oscillations. Theoretical analysis indicates that the primary cause is the excessively short duration of the deceleration phase in the stopping area. In addition, because the rotor is an elastic component, the sharp decrease in rotational speed generates considerable friction, causing the rotor—made of a thin hard aluminum plate—to undergo torsional deformation along its axis and experience substantial torsional vibrations in the stopping area. To verify the rationality of the above mechanism analysis, the following section conducts kinematic and dynamic simulation analysis of the motor model using ADINA software based on the microstepping driving method. The following simulations will sequentially conduct dynamic analysis of key points, kinematic and force analysis of the stator-friction interface, and finally systematically investigate the complete motor deformation, interface stress, friction force, and output performance.

##### Dynamic Simulation of Point W on the Friction Material

**Figure 21:**
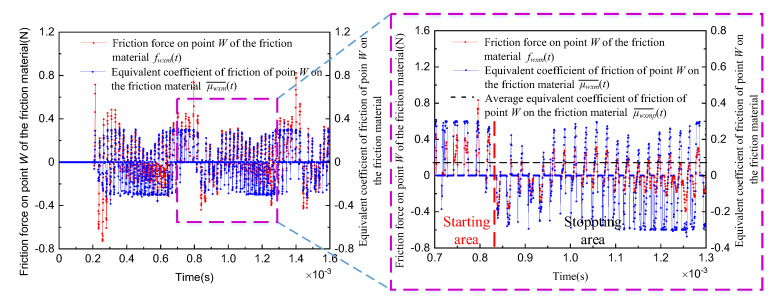
Analysis diagram of friction force and coefficient of friction for point W on the friction material under excitation of the microstepping driving method.

As shown in Figure 21, under microstepping driving, the stator of the ultrasonic motor is in a traveling wave vibration state in the starting area, where the points on the stator surface perform clockwise elliptical motion to drive the rotor rotation through friction. Upon entering the stopping area, the traveling wave vibration of the stator weakens, and the stator and rotor friction material enter a strong contact state. The contact area increases significantly, the normal contact force rises, and a large friction force is generated, effectively suppressing the rotational motion of point *W*. Consequently, the direction of the friction force on point *W* of the friction material changes periodically with the alternation between the starting and stopping areas (indicated by the pink box in Figure 21). In addition, the high-frequency vertical vibration of stator point *Q* causes the rotor to “float”, leading to intermittent contact and separation between point *W* and point *Q*, and thus an intermittent friction force. By combining Equations (17)–(19), the instantaneous tangential friction force $f_{wxm}\left(t\right)$, the equivalent coefficient of friction $\bar{\mu_{wxm}}\left(t\right)$, and its average value $\bar{\mu_{wxm}}\left(t\right)$ for point *W* can be obtained. Substituting the motion simulation data of the point into Equation (19) yields an average equivalent coefficient of friction $\bar{\mu_{wxmp}}$= 0.008.

##### Simulation Analysis of Stator Deformation and Contact Interface Stress of the Friction Material in the Complete Motor Model

(1) Simulation Analysis of Stator Deformation and Contact Interface Stress on the Friction Material for the Complete Motor Model

**Figure 22:**
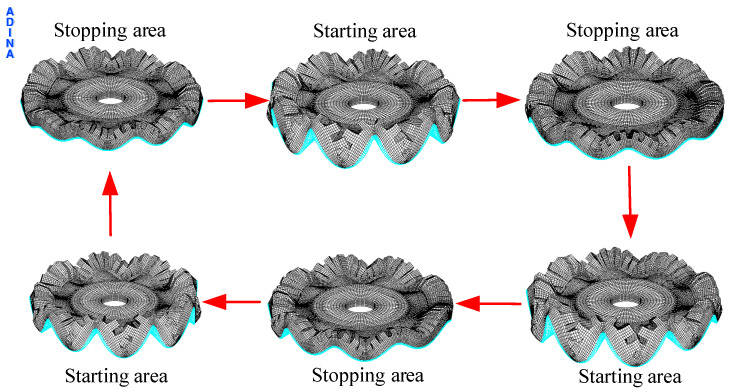
Schematic diagram of stator deformation under excitation of the microstepping driving method.

As shown in Figure 22, when the motor model is based on the microstepping driving method, the nine wave crests excited on the stator surface have uniform amplitudes in the starting area. They remain in continuous contact with the friction material in the form of a traveling wave and rotate around the axis. In the stopping area, under the applied preload, the amplitudes of the nine wave crests decrease rapidly. When the amplitude drops to a certain value, both the crests and troughs on the stator surface come into contact with the friction material, resulting in a sharp increase in the contact area. According to the friction binomial law, the friction force between the stator and the friction material also increases sharply. Since the preload distribution on the friction material surface reflects the friction force distribution acting on the rotor, the following analysis will be carried out using the stress analysis diagram of the contact surface between the stator and the friction material.

**Figure 23:**
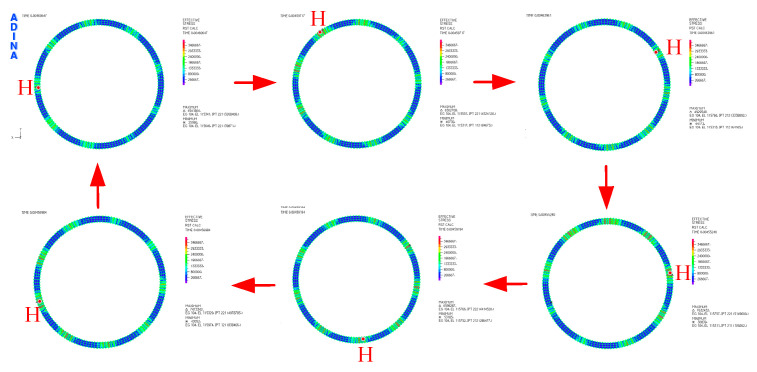
Stress distribution diagram of the stator-friction material contact interface under excitation of the microstepping driving method.

To analyze the variation in contact interface area under the microstepping driving method and its influence on motor output performance, simulation analysis of the contact interface was conducted using the stator deformation and contour plot functions of ADINA software. The results are presented in Figure 23. With this driving method, the two phases of the stator are simultaneously energized and de-energized in the starting area and stopping area. The amplitudes of the nine wave crests on the stator remain essentially consistent, and the preload distribution is relatively uniform.

**Figure 24:**
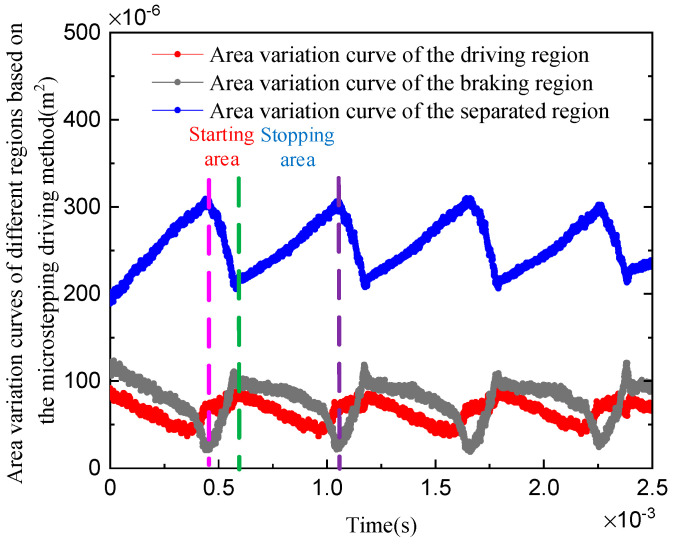
Area distribution diagrams of the starting area and stopping area based on the microstepping driving method.

As shown in Figure 24, after the motor speed stabilizes, the areas of the driving region (red curve), braking region (black curve), and separated region (blue curve) exhibit periodic wave-like variations. The areas of the driving and braking regions alternately increase and decrease, with their area difference fluctuating synchronously. The separated region consistently maintains an area significantly larger than the other two regions, with its variation also following a wave-like pattern. This alternating area variation directly determines the dynamic characteristics of the interface friction force. When the driving region dominates, acceleration torque prevails, resulting in motor acceleration; when the braking region dominates, deceleration torque prevails, leading to motor deceleration; and when the separated region dominates, no effective driving force is present, maintaining only a contact–separation state. Consequently, both the instantaneous interface friction force and the equivalent coefficient of friction exhibit wave-like variations, with the friction force direction periodically reversing as the two regions alternately take dominance.

Compared with Figure 18 (superimposed pulse driving method), the microstepping driving method, subjected to alternating energization and de-energization, results in substantially larger fluctuations in the areas of the driving and braking regions during the starting and stopping phases. This dynamic interface behavior directly causes more pronounced speed oscillations and a considerably higher speed fluctuation rate.

(2) Simulation Analysis of Friction Force and Coefficient of Friction at the Friction Material Interface

**Figure 25:**
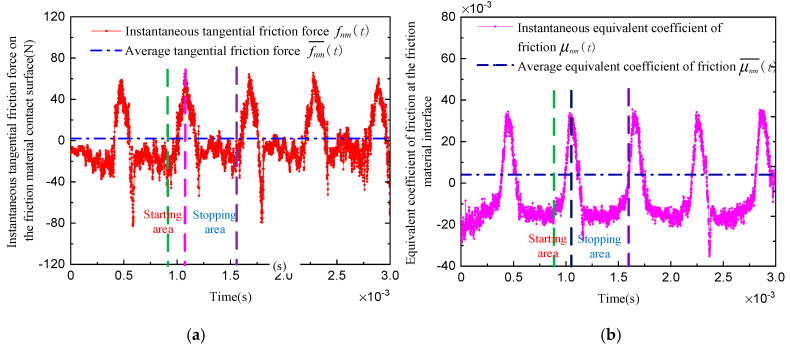
Analysis of friction forces and friction coefficients at the friction material interface based on the microstepping driving method. (**a**) Instantaneous and average friction forces at the interface of friction material; (**b**) Instantaneous and average equivalent coefficients of friction at the friction material interface.

After the motor speed stabilizes, the time-dependent variations in the instantaneous tangential friction force $f_{nm}(t)$ at the friction material contact surface and the average tangential friction force $\bar{f_{nm}}(t)$ over $N_{jm}$ cycles of elliptical motion are presented in Figure 25a. Figure 25b illustrates the time-dependent variations in the instantaneous equivalent friction coefficient $\mu_{nm}(t)$ for all points at the friction material interface and the average equivalent friction coefficient $\bar{\mu_{nm}}(t)$ over $N_{jm}$ cycles of elliptical motion.

According to Equation (29), the average tangential friction force at the interface of the friction material over $N_{jm}$ cycles of elliptical motion is $\bar{f_{nm}}$ = 1.98 N. According to Equation (31), the average equivalent coefficient of friction at the friction material interface over $N_{jm}$ cycles of elliptical motion is $\bar{\mu_{nm}}$ = 0.004. To further reveal the overall operational performance of the motor under the microstepping driving method, output performance simulation analysis is carried out based on the complete motor finite element model, with emphasis on the dynamic response characteristics of torque and speed.

(3) Simulation Analysis of Output Performance of the Complete Motor Model

**Figure 26:**
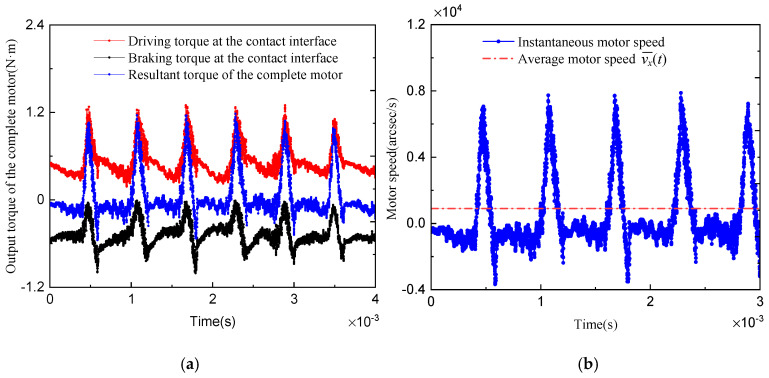
Simulation analysis diagram of motor torque output and speed based on the microstepping driving method. (**a**) Simulation of motor torque output; (**b**) Simulation of motor speed.

Based on Equation (32), the simulation curves of torque in the three regions and motor speed are presented in Figure 26a. The simulation results indicate that the motor output torque exhibits significant periodic oscillations and fluctuations. The target motor speed was set to ${v_{tar}\left(t\right)=9\times 10}^{2}$ arcsec/s. Under the same preload and load conditions, the simulation curve of motor speed under the microstepping driving method obtained using ADINA software is shown in Figure 26b. The speed exhibits periodic oscillations over time, with an average simulated speed of ${\bar{v_{x}}\left(t\right)=9.02\times 10}^{2}$ arcsec/s, which is in good agreement with the target speed. The calculated speed fluctuation rate is 228%.

## 4. Analysis of Vibration and Measurement Experiments

### 4.1. Analysis of Stator Modal Tests

**Figure 27:**
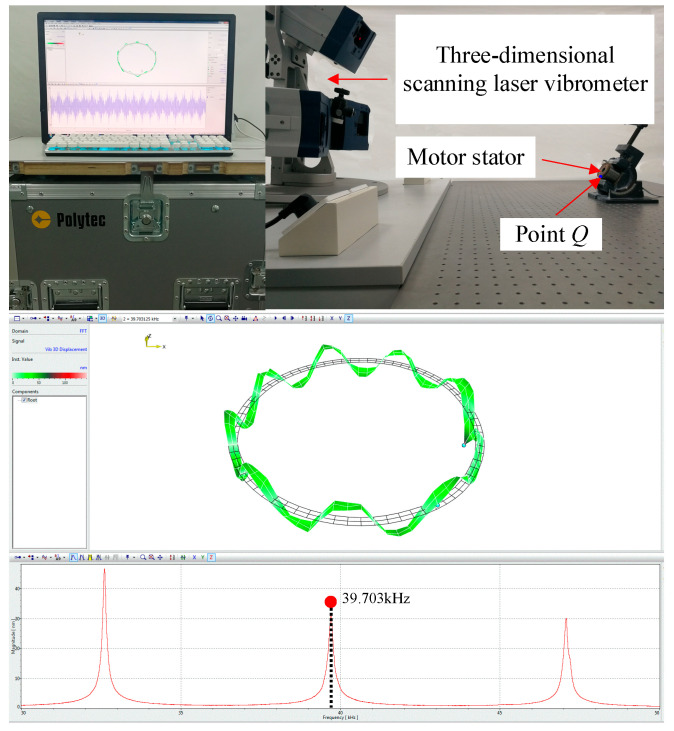
Experimental modal analysis of the stator.

To verify the accuracy of the simulation results, the 9th-order out-of-plane modal shape of the stator was measured at a voltage of 80 Vp-p using a high-frequency scanning laser vibrometer system (model PSV-500-3D) from Polytec (Germany) [29], as shown in Figure 27. The measured modal frequency from the sweep test is $\omega_{n0}=39.703$ kHz, which is close to the natural frequency obtained from the modal simulation using ADINA software (as shown in Figure 12), thereby confirming the validity of the stator modal simulation results.

### 4.2. Experimental Study of Stator Modal Vibration Measurement Based on Two Driving Methods

**Figure 28:**
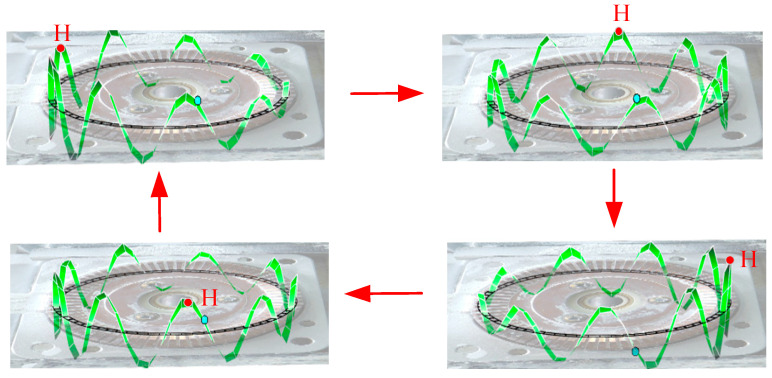
Stator vibration mode diagram based on the superimposed pulse driving method.

**Figure 29:**
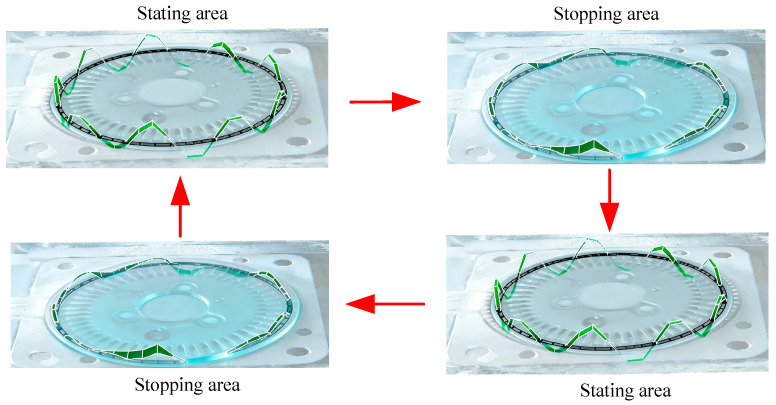
Stator vibration mode diagram based on the microstepping driving method.

To verify the accuracy of the stator deformation contour plots generated by ADINA software, the parameters listed in Table 1 and Table 2 were first entered into Tektronix ArbExpress software (version 1.2.0, released in 2021) to generate waveform files. These files were then imported into a signal generator, and the signals were output to a 3D laser vibrometer. Finally, under an excitation voltage of 80 V_p__-__p_, the stator vibration mode diagrams corresponding to the superimposed pulse driving method and the microstepping driving method were measured, as shown in Figure 28 and Figure 29, respectively.

### 4.3. Experimental Study of Stator Modal Vibration Measurement Using the Superimposed Pulse Driving Method

As shown in Figure 28, the stator vibration mode test was conducted without rotor assembly, as the presence of the rotor would obstruct the vibration mode measurement. After excitation using the superimposed pulse driving method, nine wave crests with uneven amplitude distribution were formed on the stator surface. Only one wave crest exhibited the maximum amplitude, while the amplitudes of the remaining crests decreased progressively on either side of this peak. This wave crest propagated around the stator axis in the form of a traveling wave. The overall vibration pattern was found to be in good agreement with the vibration mode obtained from the simulation model (as shown in Figure 16), thereby fully validating the accuracy of the simulation model in this study.

Combined with the preceding simulation analysis, under operating conditions with the rotor assembled and preload applied, the wave crest with the maximum amplitude forms the largest contact area with the friction material and plays the dominant driving role for the rotor. In contrast, the remaining wave crests with smaller amplitudes exhibit weak contact with the rotor and are unable to generate effective driving.

### 4.4. Experimental Study of Stator Modal Vibration Measurement Using the Microstepping Driving Method

When excited by the microstepping driving method, the stator vibration mode test was conducted, and the results are shown in Figure 29. The test results indicate that in the starting area, nine traveling wave crests with uniform amplitudes are excited on the stator surface, with each crest rotating around the stator axis. In the stopping area, the amplitudes of the stator wave crests decrease rapidly. This variation pattern of the vibration mode is generally consistent with the stator deformation obtained from the simulation model (as shown in Figure 22), thereby validating the reasonableness of the simulation model in this study. Combined with the simulation analysis, under operating conditions with the rotor assembled and preload applied, when the stator amplitude in the stopping area decreases to a certain level, both the wave crests and troughs come into contact with the friction material, resulting in a sharp increase in the contact area. According to the friction binomial law, the friction force between the stator and the friction material also increases sharply. Both the vibration mode test and simulation analysis indicate that the periodic and drastic variations in stator vibration mode, interface contact state, and friction force are the core reasons for the significant periodic speed oscillations of the motor under microstepping driving.

## 5. Experiment Research

### 5.1. The Establishment of Experimental Platform

**Figure 30:**
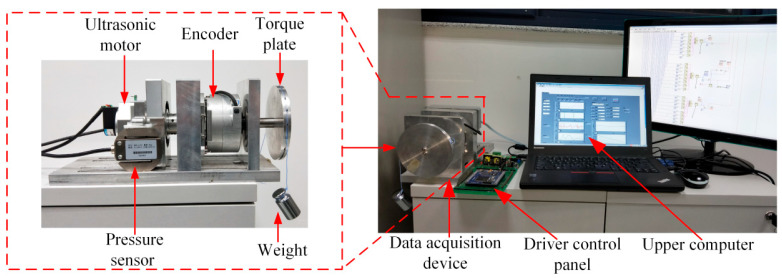
Configuration of the experiment platform.

As shown in Figure 30, the experimental platform consists of a data acquisition device, a drive control board, and an upper computer. In this platform, a high-precision encoder is employed to measure the low speed of the motor. As an important feedback component for servo system motors, the encoder can feed back the measured linear displacement, angular displacement, and speed to the control system. Specifically, a 25-bit hollow-shaft absolute encoder manufactured by Heidenhain, based on the EnDat bidirectional data interface (model ECN225), is used, with a position count of 33,554,432 per revolution. In addition, a string is used to drag the weight on the outer ring of the torque plate, and the load torque is obtained as the product of the pressure measured by the pressure sensor and the radius of the rotor. Furthermore, the host computer test system is based on a LabVIEW program, which sends control commands to the DSP and receives data for real-time display and storage.

### 5.2. Block Diagram of the Test System

**Figure 31:**
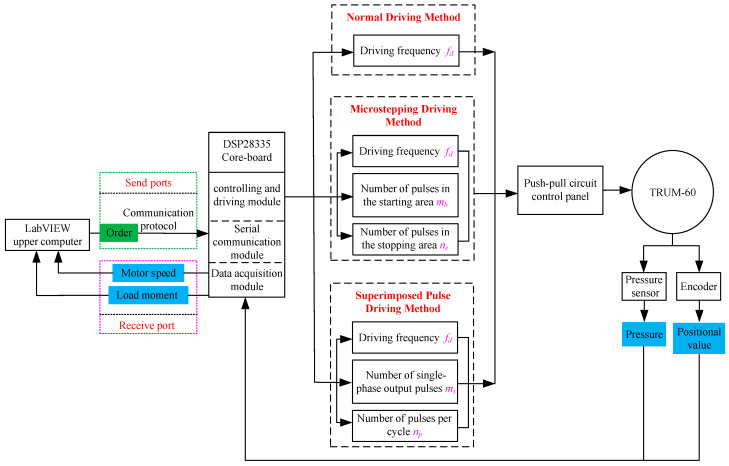
Block diagram of the motor speed testing system.

Figure 31 presents the framework of the ultrasonic motor performance test system built in this paper. The system provides a unified and standardized test platform for the comparative experiments of three driving methods: the normal driving method, the superimposed pulse driving method, and the micro-step driving method. The overall system adopts a complete closed-loop architecture comprising host computer human–machine interaction, core controller computation and processing, power driving amplification, motor execution, and status data feedback acquisition. The core hardware includes a LabVIEW host computer, a DSP28335 core board (integrating three functional modules: serial communication, control and drive, and data acquisition), a push-pull circuit drive control board, a TRUM-60 ultrasonic motor, as well as two types of signal acquisition elements: a pressure sensor and an encoder.

The complete system workflow is as follows: After setting the test conditions and driving modes on the LabVIEW host computer, corresponding control commands are generated and sent to the DSP28335 core board via serial communication protocol. Upon parsing the commands, the control and drive module invokes the independent speed control logic of the corresponding driving mode to generate matching control signals. These signals are power-amplified by the push-pull circuit drive control board, and then drive the TRUM-60 ultrasonic motor to operate stably according to the preset strategy. During motor operation, the pressure sensor and encoder collect the motor preload, rotor position, and real-time speed signals in real time. All collected data are sent back to the data acquisition module of the core board, and finally uploaded to the LabVIEW host computer for data storage, display, and subsequent analysis, thus forming a complete control and testing closed loop. The specific control parameter settings for the three driving modes are detailed in Table 1 and Table 2.

### 5.3. Comparative Experiments Based on the Normal Driving Method and the Superimposed Pulse Driving Method

**Figure 32:**
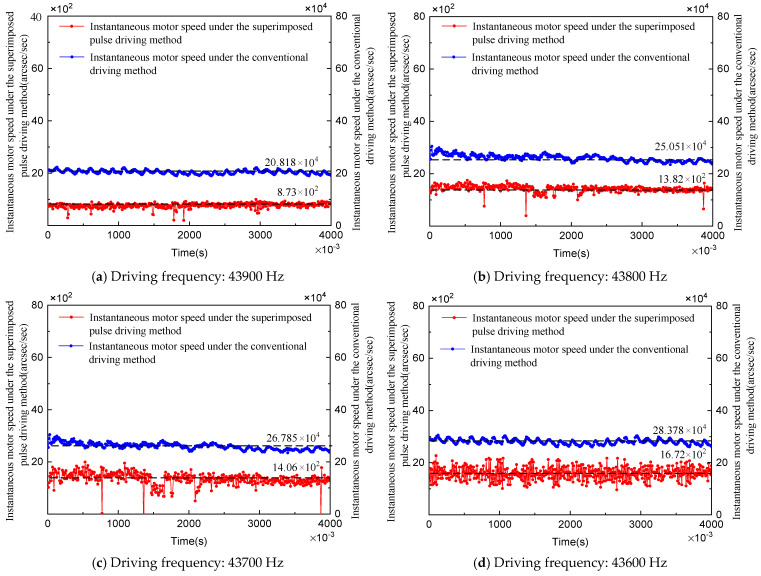
Experimental diagram of motor speed at different driving frequencies based on two driving methods.

When the normal driving method is used to drive the motor, the motor speed is typically regulated by adjusting the driving frequency. According to the simulation conclusions of the superimposed pulse driving method, at the same driving frequency, the motor speed under the superimposed pulse driving method is significantly lower than that under the normal driving method. To verify the correctness and reliability of the simulation conclusions and the simulation model, further comparative open-loop control experiments are conducted in this paper to validate the above simulation results. The specific control parameters of the superimposed pulse driving method are set according to Table 1. To ensure complete consistency of boundary conditions between the simulation and the experiment, and to guarantee the rigor of the comparative experiments while eliminating interference from non-driving factors, the motor preload is uniformly set to 180 N and the load is uniformly set to 0.2 N under all test conditions. The key operating parameters are kept strictly consistent with those in the simulation system. Four driving frequency points are selected for the experiments: 43,600 Hz, 43,700 Hz, 43,800 Hz, and 43,900 Hz. The experiments are conducted under open-loop control throughout. In the following sections, four sets of open-loop motor speed experiments are carried out by changing the driving frequency using both the normal driving method and the superimposed pulse driving method. The experimental results after the motor speed stabilizes are shown in Figure 32.

The experimental results show that as the driving frequency decreases, the motor speed under both the normal driving method and the superimposed pulse driving method exhibits an increasing trend. Moreover, at all tested frequency points, the motor speed under the superimposed pulse driving method is significantly lower than that under the normal driving method, with a difference of up to two orders of magnitude. This experimental trend is highly consistent with the simulation conclusions derived earlier for the superimposed pulse driving method. The core reason lies in the inherent driving characteristics of the superimposed pulse driving method: stator points undergo periodic alternating clockwise and counterclockwise rotational motion, and the motor adopts a single-peak driving mode. Among the nine wave crests on the stator, only the single high-amplitude wave crest plays the dominant driving role, while the remaining wave crests exhibit either weak contact or no effective contact. The overall driving characteristic is dominated by a single point with multiple points contributing only weakly, which greatly limits the upper limit of motor speed from the driving source. In particular, at the test frequency of 43,900 Hz, the simulated motor speed under the superimposed pulse driving method is very close to the experimental speed, fully demonstrating the reference value of the simulation results and confirming the reliability of the simulation model. The experimental results are in full agreement with the previous simulation conclusions, effectively validating the correctness of the beat traveling wave theory underlying the superimposed pulse driving method, and laying a solid foundation for subsequent dynamic simulation analysis of stator points.

The superimposed pulse driving scheme proposed in this paper is primarily oriented towards the ultra-low-speed precision control application scenario of semiconductor packaging equipment. The design prioritizes ensuring low-speed running smoothness and control accuracy as its core objectives, rather than pursuing high motor speed output. With this scheme, the maximum motor speed is reduced, and the corresponding peak output power and maximum output torque also decrease. However, in such ultra-low-speed working conditions as semiconductor precision packaging, there is no practical demand for high-speed motor performance; instead, stringent requirements are placed on low-speed running smoothness and position control accuracy. Therefore, this design trade-off—sacrificing some maximum speed in exchange for low-speed control accuracy and running smoothness—is fully justified and engineeringly necessary.

### 5.4. Test Experiments and Analysis of Results Based on Two Drive Methods

**Figure 33:**
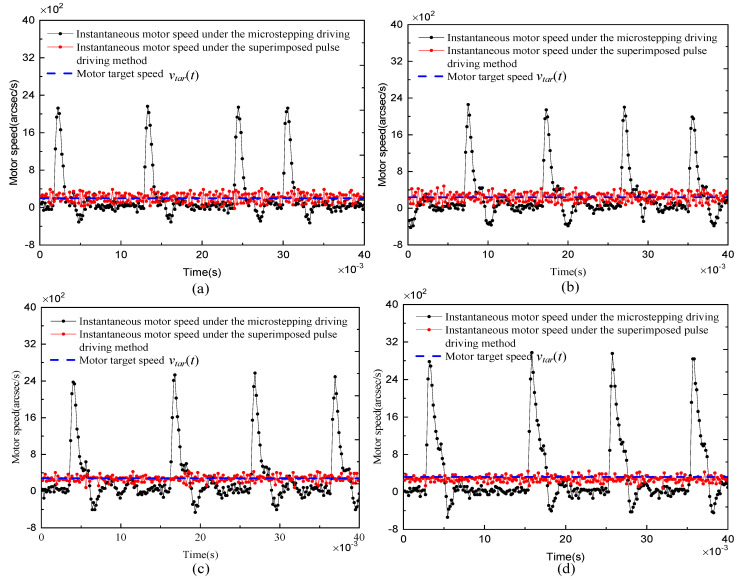
(**a**–**d**) Motor speed curves under two driving methods at different target speeds (200, 240, 280, and 320 arcsec/s, respectively).

The PID control algorithm is one of the most widely used closed-loop control techniques in industrial control due to its simple principle, strong robustness, and ease of engineering implementation [30]. In this paper, the trial-and-error method is employed to tune the PID parameters [31], and closed-loop speed regulation is achieved by adjusting the driving frequency. Four different target speeds are set. The operating parameters such as motor preload and load are kept consistent with those in the previous experiments. The motor is controlled using the microstepping driving method and the superimposed pulse driving method, respectively. The speed test results are shown in Figure 33.

As shown in Figure 33, when the target motor speeds were set to 200, 240, 280, and 320 arcsec/s, the motor speed under the microstepping driving method exhibited significant large-amplitude periodic oscillations. The speed fluctuation rates of the motor corresponding to the two driving methods were calculated using Equation (37) in Ref. [16], and the results are presented in Table 3. The four sets of comparative data indicate that, at the same target speed, the speed fluctuation rate of the superimposed pulse driving method is markedly lower than that of the microstepping driving method.

A small deviation exists between the experimentally measured speed fluctuation rates and the simulation results. This discrepancy is primarily attributable to the fact that the simulation model was established under ideal conditions, without incorporating external interference factors such as ambient temperature and humidity. Nevertheless, the trend that the superimposed pulse driving method achieves a significantly lower speed fluctuation rate than the microstepping driving method at identical target speeds is fully consistent with the simulation conclusions. These results not only validate the accuracy of the simulation model presented in this study but also demonstrate that the proposed model can faithfully reflect the actual driving characteristics of the motor, showing excellent agreement with the real friction behavior at the stator-rotor contact interface. Thus, the model possesses strong engineering practicality and research value as an alternative to direct experimental measurements.

## 6. Conclusions

Most theoretical and simulation studies of traditional ultrasonic motors have adopted a macroscopic mechanical perspective, treating the motor as a single mechanical system for kinematic, dynamic, and equivalent circuit modeling. Such approaches involve extensive linearization and idealization of the system, allowing only preliminary mechanism analysis and a general grasp of performance trends, but failing to achieve precise quantitative analysis. Once quantitative calculations are performed, the results often deviate to some extent from actual operating conditions. Moreover, these studies overlook critical factors such as the microstructure of contact surfaces, preload distribution, and vibration mode transmission, making it difficult to capture the true characteristics of ultrasonic motors—including strong nonlinearity, multiphysics coupling, and complex interface behavior—which further leads to a mismatch between theoretical calculations and experimental results. In response to the above research limitations, the present study focuses on two core aspects.

On the one hand, the simulation model developed in this paper fully accounts for the strong nonlinear characteristics and multiphysics coupling effects of the motor, effectively addressing the aforementioned shortcomings of traditional simplified methods. Although minor discrepancies exist between the simulation data and experimental test results due to environmental disturbances such as temperature and humidity under actual operating conditions, the model remains capable of accurately revealing the complex interface-driven mechanism of the motor. On the other hand, owing to the scarcity and high cost of instruments that can precisely measure the friction force at the stator-rotor interface, it is difficult to elucidate the underlying mechanism through direct measurement. Therefore, this study relies on simulation analysis, complemented by modal testing and experimental research for cross-validation, to fully verify the correctness and rationality of the proposed model, thereby ensuring its reliable engineering applicability and research value as an alternative to direct measurement.

Based on the above model and experimental platform, comparative experiments between the normal driving method and the superimposed pulse driving method further indicate that the fundamental reason why the superimposed pulse driving method can achieve ultra-low-speed operation lies in its inherent driving characteristics: stator points undergo periodic alternating clockwise and counterclockwise rotational motion, and the motor adopts a single-peak driving mode. Among the nine wave crests on the stator, only the single high-amplitude wave crest plays the dominant driving role, while the remaining wave crests exhibit either weak contact or no effective contact. The overall driving characteristic is dominated by a single point with multiple points contributing only weakly, which greatly limits the upper limit of motor speed from the driving source, making it inherently suitable for ultra-low-speed conditions. On this basis, this paper systematically compares the performance differences between the superimposed pulse driving method and the microstepping driving method under ultra-low-speed conditions. Simulation and experimental results show that the superimposed pulse driving method, through the mechanism of single-peak dominance and smooth alternation between the driving and braking zones, can significantly reduce the speed fluctuation rate from 228% (of microstepping driving) to 32%, confirming that the superimposed pulse driving method designed on the basis of the beat traveling wave theory fundamentally overcomes the inherent shortcomings of the traditional microstepping driving method. The integrated research approach of “simulation + vibration measurement + experiment” established in this paper provides new methods and references for investigating the driving characteristics and underlying mechanisms of similar motors.

## Figures and Tables

**Table 1:** Parameter settings based on the superimposed pulse driving method.

Control Variables	Variable Value
Driving frequency $f_{d}$	43,900 Hz
Number of single-phase output pulses $m_{s}$	12
Number of pulses per cycle $n_{p}$	20

**Table 2:** Parameter settings based on the microstepping driving method.

Control Variables	Variable Value
Driving frequency $f_{d}$	43,900 Hz
Number of pulses in the starting area $m_{b}$	6
Number of pulses in the stopping area $n_{e}$	28

**Table 3:** Comparison of motor speed fluctuation rates between two driving methods.

Target Motor Speed (arc sec/sec)	Speed Fluctuation Rate Under the Microstepping Driving Method	Speed Fluctuation Rate Under Superimposed Pulse Driving Method
200	257.7%	47.3%
240	217.5%	41.0%
280	212.9%	33.5%
320	229.5%	23.8%

## Data Availability

The original contributions presented in this study are included in the article. Further inquiries can be directed to the corresponding author.
